# Genetic diversity and population structure of modern wheat (*Triticum aestivum* L.) cultivars in Henan Province of China based on SNP markers

**DOI:** 10.1186/s12870-023-04537-9

**Published:** 2023-11-04

**Authors:** Wenjing Tang, Zhongdong Dong, Lifeng Gao, Xicheng Wang, Tianbao Li, Congwei Sun, Zongli Chu, Dangqun Cui

**Affiliations:** 1 College of Agronomy/Collaborative Innovation Center of Henan Grain Crops, Henan Agricultural University, Zhengzhou, 450046 China; 2 Henan Agricultural Remote Sensing Monitoring Center, Zhengzhou, 450002 China; 3 Institute of Crop Sciences, Chinese Academy of Agricultural Sciences, Beijing, 100081 China; 4 Henan Academy of Agricultural Sciences, Zhengzhou, 450002 China; 5 College of Agronomy, Xinyang Agriculture and Forestry University, Xinyang, 464000 China

**Keywords:** Wheat, Genetic diversity, Population genetic structure, Principal component analysis, Single-nucleotide polymorphism

## Abstract

**Background:**

Henan is the province with the greatest wheat production in China. Although more than 100 cultivars are used for production, many cultivars are still insufficient in quality, disease resistance, adaptability and yield potential. To overcome these limitations, it is necessary to constantly breed new cultivars to maintain the continuous and stable growth of wheat yield and quality. To improve breeding efficiency, it is important to evaluate the genetic diversity and population genetic structure of its cultivars. However, there are no such reports from Henan Province. Therefore, in this study, single nucleotide polymorphism (SNP) markers were used to study the population genetic structure and genetic diversity of 243 wheat cultivars included in a comparative test of wheat varieties in Henan Province, aiming to provide a reference for the utilization of backbone parents and the selection of hybrid combinations in the genetic improvement of wheat cultivars.

**Results:**

In this study, 243 wheat cultivars from Henan Province of China were genotyped by the Affymetrix Axiom Wheat660K SNP chip, and 21 characteristics were investigated. The cultivars were divided into ten subgroups; each subgroup had distinct characteristics and unique utilization value. Furthermore, based on principal component analysis, Zhoumai cultivars were the main hybrid parents, followed by Aikang 58, high-quality cultivars, and Shandong cultivars. Genetic diversity analysis showed that 61.3% of SNPs had a high degree of genetic differentiation, whereas 33.4% showed a moderate degree. The nucleotide diversity of subgenome B was relatively high, with an average π value of 3.91E-5; the nucleotide diversity of subgenome D was the lowest, with an average π value of 2.44E-5.

**Conclusion:**

The parents used in wheat cross-breeding in Henan Province are similar, with a relatively homogeneous genetic background and low genetic diversity. These results will not only contribute to the objective evaluation and utilization of the tested cultivars but also provide insights into the current conditions and existing challenges of wheat cultivar breeding in Henan Province, thereby facilitating the scientific formulation of breeding objectives and strategies to improve breeding efficiency.

**Supplementary Information:**

The online version contains supplementary material available at 10.1186/s12870-023-04537-9.

## Background

Henan is the province with the greatest wheat production in China, with an annual cultivated area of more than 5.69 million ha, accounting for approximately 24.8% of the national planting area and 28.3% of the national yield (http://www.stats.gov.cn/tjsj/zxfb/202107/t20210714_1819380.html; http://hazd.stats.gov.cn/zxfbyjd/show-394.html). Although there are more than 100 cultivars planted for crop production, many remain insufficient in quality, disease resistance, adaptability, and yield potential. Moreover, due to the interference of natural or human factors such as selection, mutation, migration and genetic drift, the characteristics of cultivars may change, and the appropriate planting time of a cultivar during production is limited. Therefore, it is necessary to continuously breed new cultivars to overcome these limitations, cope with and meet changing natural and production conditions, and maintain a continuous, stable increase in wheat yield. However, to improve breeding efficiency and accelerate the breeding process, it is necessary to characterize population genetic structure and genetic diversity [1–3].

Population genetic structure refers to the distribution of and relationship among various genotypes or genes in a population, whereas genetic diversity refers to the sum of genetic information carried by all organisms on Earth and generally refers to intraspecific genetic diversity, that is, the sum of genetic variation among individuals or the same species or in a group. Over the years, breeders and geneticists have performed extensive research on the population structure and genetic diversity of wheat, corn, and other crops [4–8]. However, with advances in molecular biology technology, research on population structure has shifted from phenotypic to molecular biology-based [9–14]. Different software programs, including STRUCTURE [15, 16], ADMIXTURE [17], MEGA [18, 19] POWERMARKER [20], GenAlEx [21], PLINK [22], and GENEPOP [23], have been developed to assist researchers in the analysis of the genetic composition, differentiation, classification, and diversity of populations.

Previous studies have examined the genetic diversity and population structure of Urartu wheat (*Triticum urartu*) [24], *Aegilops tauschii* [25], crested wheatgrass (*Agropyron cristatum* L.) [26], wild emmer wheat (*Triticum dicoccoides*) [27], durum wheat (*Triticum durum*) [28–31], synthetic wheat [32], and common wheat (*Triticum aestivum*) [3, 33–41] using simple sequence repeats (SSR), diversity array technology (DArT), and single-nucleotide polymorphisms (SNPs), as well as other molecular markers and analysis software. Although studies from around the world have investigated the population genetic structure and genetic diversity of wheat, no such reports have been provided for Henan Province. Based on these findings, this study was designed to examine the population genetic structure and genetic diversity of 243 wheat cultivars included in the Comparative Test of Wheat Varieties in Henan Province using the Affymetrix Axiom Wheat660K SNP chip with broad application prospects [42, 43], aiming to provide a reference for the utilization of backbone parents and the selection of hybrid combinations in wheat cultivar genetic improvement.

## Results

### Estimates of Fst and π of modern wheat cultivars in Henan Province

**Fig. 1:**
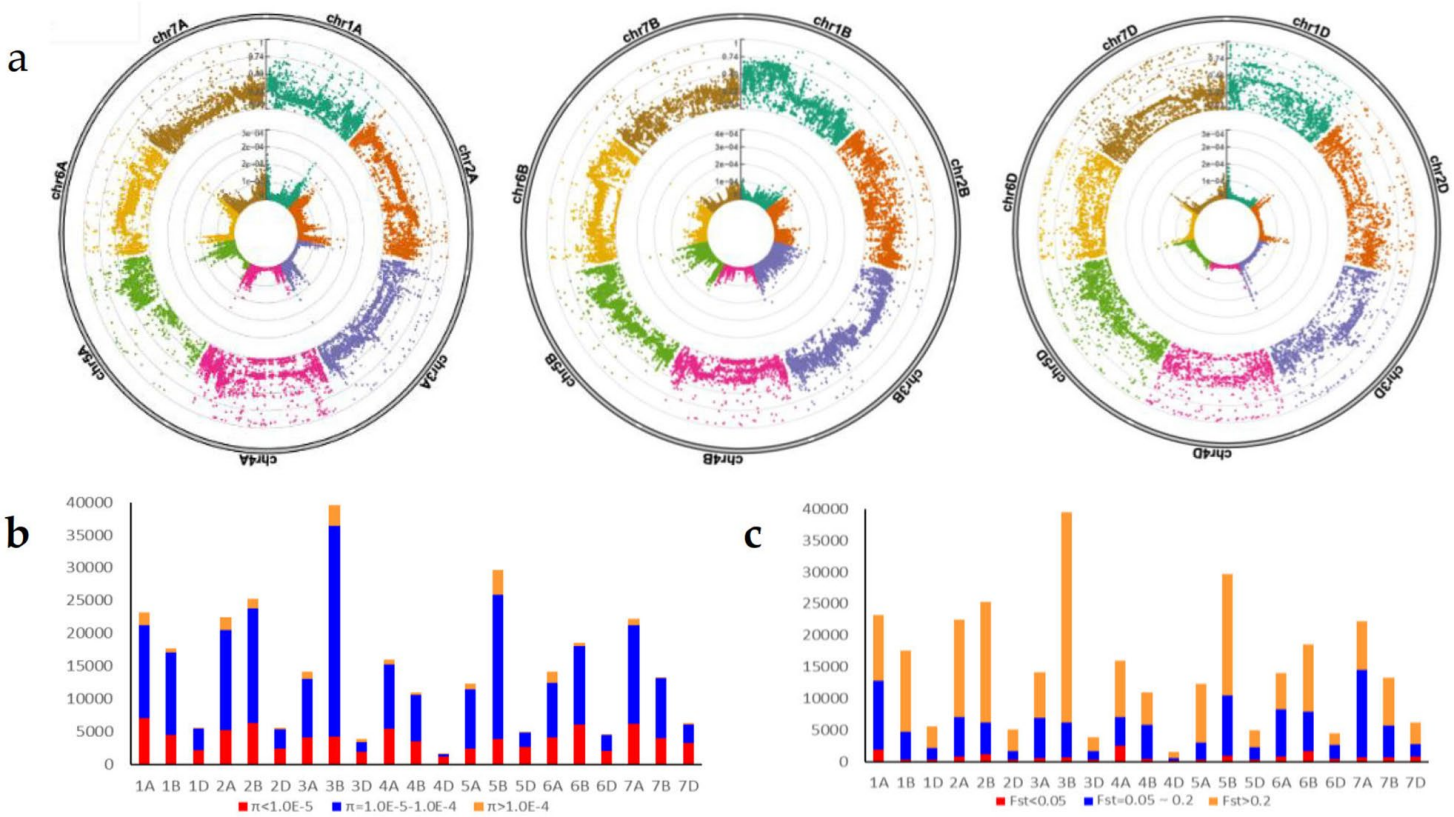
The distribution and comparisons of genetic diversity parameters Fst and π in each subgenome and its chromosome. **a** Distribution of Fst (outer circle) and π (inner circle) on different chromosomes of three subgenomes. **b** Comparisons of SNP numbers of Fst at different levels in different chromosomes. **c** Comparisons of SNP numbers of π at different levels in different chromosomes

Figure 1a shows the distribution of the genetic diversity parameters Fst and π in each subgenome and its chromosomes. In population genetics, the genetic differentiation coefficient (Fst) is used to measure the degree of differentiation among populations, where the higher the Fst value is, the greater the genetic distance. Figure 1b shows the number of SNPs on each chromosome (21 chromosomes) at different Fst levels. The numbers of SNPs with low (Fst < 0.05), moderate (Fst = 0.05 ~ 0.2), and high (Fst > 0.2) genetic differentiation were 16,496 (5.3%), 103,962 (33.4%), and 190,576 (61.3%), respectively. Further analysis showed that for subgenome A, the percentages of SNPs with low, medium and high differentiation were 6.3%, 41.7% and 52.0%, respectively; for subgenome B, they were 3.8%, 26.5% and 69.7%; and for subgenome D, they were 8.7%, 34.7% and 56.6%. The Fst means of subgenomes A, B, and D were 0.22, 0.26, and 0.24, respectively.

Additionally, π is a measure of nucleotide diversity in a population. A larger π indicates higher nucleotide diversity, whereas a lower π specifies lower nucleotide diversity between the two loci. Figure 1c shows the number of SNPs on each chromosome at different π levels. The numbers of SNPs with relatively low (π < 1.0E-5), moderate (π = 1.0E-5 ~ 1.0E-4), and high (π > 1.0E-4) nucleotide diversity were 82,410 (26.4%), 208,551 (66.9%), and 20,683 (6.6%), respectively. Further analysis showed that for subgenome A, the percentages of SNPs with relatively low, moderate, and high nucleotide diversity were 27.8%, 64.8%, and 7.5%, respectively, whereas for subgenome B, they were 20.8%, 72.7%, and 6.5%, and for subgenome D, they were 48.3%, 47.5%, and 4.2%. The π means of subgenomes A, B, and D were 3.68E-5, 3.91E-5, and 2.44E-5, respectively.

### Principal Component Analysis (PCA) of modern wheat cultivars in Henan Province

**Fig. 2:**
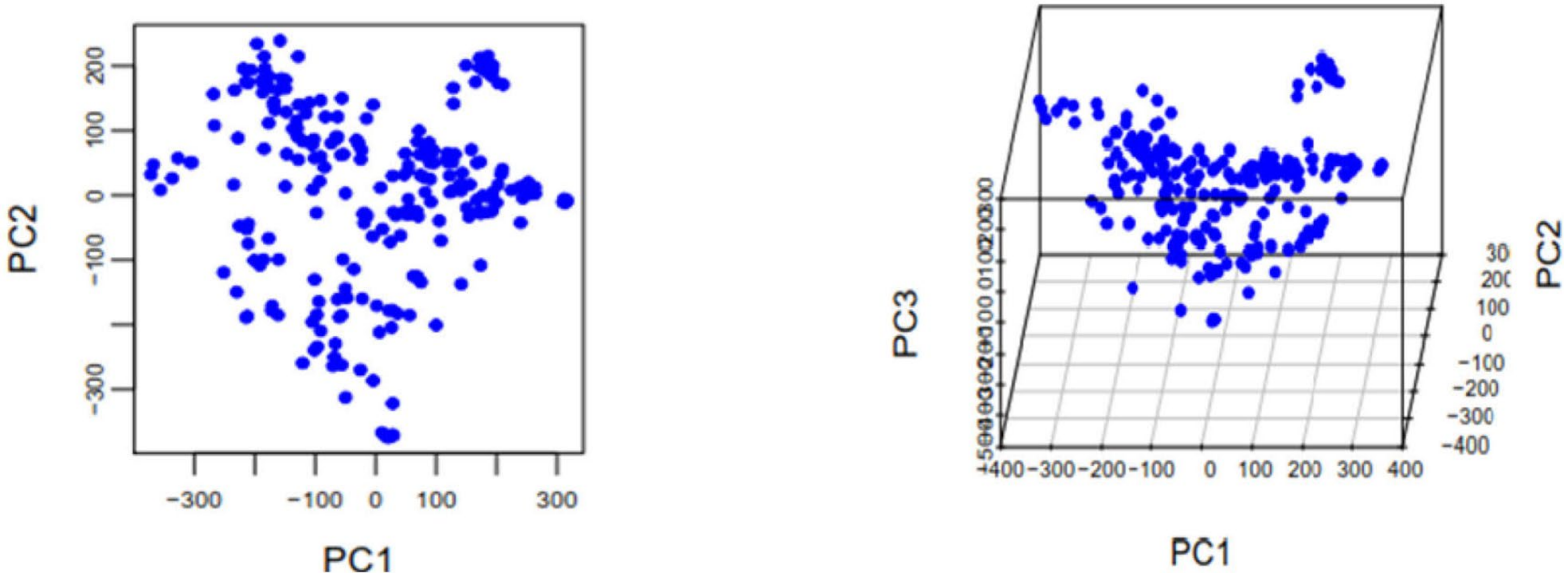
Scatter plot of first vs second and third principal components (eigenvectors) of 243 tested cultivars

The tested cultivars in this study were bred by cross-breeding of cultivars widely planted in the past and now with varieties bred abroad or in other parts of China or new materials generated by breeding units themselves. Therefore, there is a certain association between these cultivars. If the genotype information for these cultivars can be transformed into independent gene type information, insights may be provided regarding the main parent resources for breeding, thereby allowing for cross combination selection to be purposefully carried out to facilitate increased breeding efficiency. To this end, PCA of 243 tested cultivars was performed using GAPIT software [44]. Figure 2 provides a two-dimensional and three-dimensional diagram of the first three principal components (eigenvectors), demonstrating that these 243 cultivars could be divided into several subgroups.

PCA of the 243 cultivars showed that eight principal components (eigenvectors) had relative contribution rates > 2%. The relative contribution rates of principal components 1–8 were 10.15%, 8.59%, 6.41%, 3.77%, 2.99%, 2.90%, 2.55%, and 2.28%, respectively, and the cumulative contribution rate was 39.64%. Table 1 lists the top 10 elements (the absolute value of the coefficients of these elements is relatively high) of the selected principal components (eigenvector) and their corresponding cultivars.

**Table 1:** The top 10 elements of the selected principal components and their corresponding cultivars

**PC1**		**PC2**		**PC3**		**PC4**	
315.5	Zhongle 8	374.0	Hefeng 3	427.6	Shenzhou 209	− 196.3	Pingnongyan 3
315.5	Xunhe 183	373.8	Xinhuamai 818	421.2	Boyu 866	− 196.8	Jinfeng 205
314.4	Kaimai 26	373.4	Hangmai 8	419.2	Xianmai 15	− 199.6	Huimai 216
313.1	Hemai 6	372.5	Xuyan 2	311.4	Xinxuan 17	− 199.9	Jiyanmai 7
312.3	Anyumai 18	371.5	Zhengxin 758	286.4	Chuangxing 6	− 200.0	Tianlaoda 1
− 325.5	Kelinmai 969	371.0	Tianmai 119	266.6	Jinmai 1	− 202.9	Neile 268
− 335.7	Wohua 066	370.7	Meng 615	266.2	Xunmai 118	− 206.2	Hongmai 186
− 354.9	Shunmai 299	370.3	Yuyan 168	256.2	Caizhi 204	− 210.2	Chuangxing 26
− 365.5	Chuangxin 116	366.8	Xinmai 68	254.3	Dapingyuan 18	− 215.3	Zhaofeng 668
− 368.6	Zimai 615	321.9	Zhongle 9	253.1	Zhengmai 516	− 216.6	Zhengda 3087
**Eigenvalue**	24,317.4	20,572.8		15,352.6		9034.6	
**PC5**		**PC6**		**PC7**		**PC8**	
322.4	Luo 1807	183.0	SM110	332.8	Zimai 615	167.6	Shenhua 208
320.5	Zhengpinmai 24	− 174.9	Xinmai 68	289.4	Chuangxin 116	− 174.1	Xunmai 118
317.7	Shunmai 8	− 189.1	Tianmai 119	287.7	Shunmai 299	− 175.9	Jinmai 1
314.5	Jiangmai 816	− 192.1	Yuyan 168	259.1	Wohua 066	− 219.9	Gengmai 256
304.7	Lunxuan 163	− 192.7	Meng 615	222.0	Jinfeng 216	− 221.6	Haozhuangjia 1
290.2	Qiule 2126	− 193.8	Xuyan 2	213.9	Fannong 1	− 235.8	Luyan 260
277.2	TH 161	− 194.0	Zhengxin758	207.7	Weinong 208	− 258.8	Ximai 505
276.9	Jingjiumai 11	− 194.1	Hangmai 8	175.3	Kelinmai 969	− 265.4	Shengmai 102
202.0	Zhongfengmai 2	− 195.6	Hefeng 3	173.2	Luomai 166	− 305.3	Taifeng 11
188.9	Xuke 732	− 195.6	Xinhuamai 818	− 165.2	TH 161	− 305.3	Yanfeng 712
**Eigenvalue**	7153.5	6952.2		6116.0		5449.8	

In this study, all tested cultivars were bred by crossing. According to analysis of the hybrid combinations of the cultivars corresponding to the ten elements with the largest absolute coefficient values for the top 8 principal components (Table 1), the most common parents used in the cross combinations corresponding to the cultivars of PC1 and PC2 (Additional file 1: Table S1) were Zhoumai cultivars, which were used six and eight times, respectively. Additionally, the hybrid combinations corresponding to the cultivars of PC3, PC4, and PC5 included Zhoumai cultivars four, eight, and five times, respectively; Aikang 58 was used five, four, and two times, respectively; and high-quality parents (Yumai 34, Zhengmai 366, Jimai 20, Jimai 22, Zhengmai 9023, and PH82-2) were used four, two, and three times, respectively. The hybrid combinations corresponding to the cultivars of PC6 and PC7 included Zhoumai cultivars nine and six times, respectively, whereas Aikang 58 was used twice. The most commonly used parents for the hybrid combinations corresponding to the cultivars of PC8 were Shandong cultivars, seven of which were used ten times as parents. Based on the above analysis, the most important backbone parents used in wheat breeding in Henan Province are the Zhoumai cultivars, followed by Aikang 58, high-quality cultivars, and Shandong cultivars.

### Population structure of modern wheat cultivars in Henan Province

**Fig. 3:**
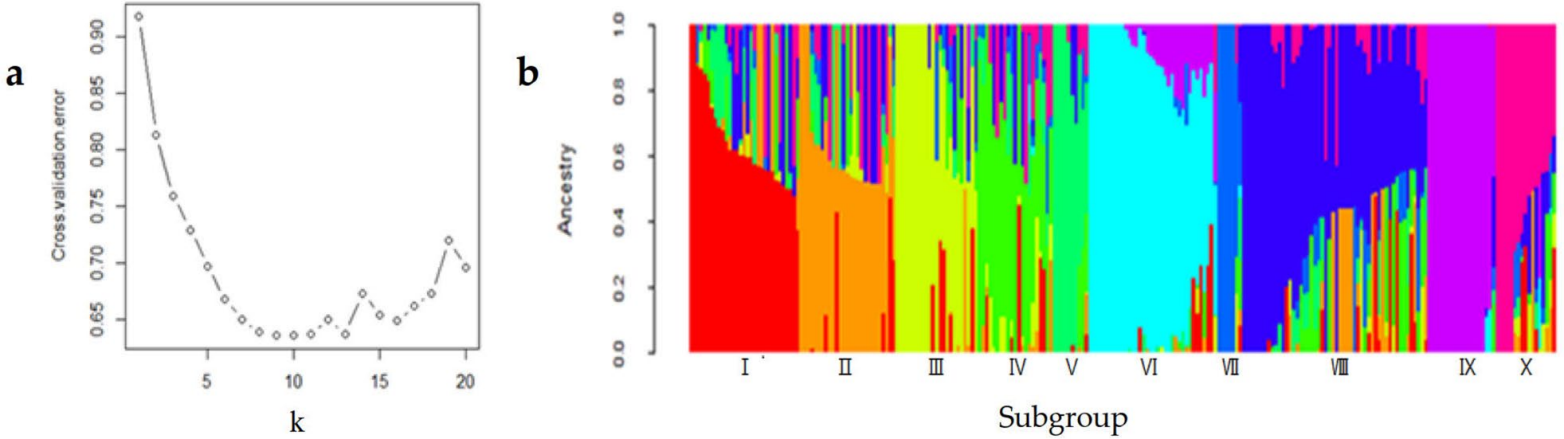
Population structure of the 243 test cultivars. **a** The plot of the scaled cross-validation error (Y-axis) with allowed ranging from 2 to 20 (X-axis); the minimum cross-validation error (0.63526) was obtained when k = 10. **b** Estimated population structure of 243 tested cultivars on k = 10, columns represent individual wheat accessions, while the length represents the proportion of each subgroup (indicated by the color) belonging to that accession

We then determined the genetic structure of the population using 395,783 high-quality SNP markers. Using ADMIXTURE software [17], the minimum cross-validation error (0.63526) was obtained when k = 10 (Fig. 3), indicating that the 243 cultivars could be divided into ten subgroups.

Analysis of variance (Table 2) showed that the expression of the 21 traits of interest was significantly affected by the test locations, whereas the expression of 20 traits was significantly affected by the genotype (subgroups). Additionally, the expression of 18 traits was significantly affected by the interaction between test locations and genotypes. Finally, the expression of 17 traits among cultivars within subgroups differed significantly.

**Table 2:** Joint analysis of variance for each trait

**Trait**	**Test locations**		**Subgroups**		**Test location × Subgroup**		**Cultivars within subgroups**		**Experimental error**
	**MS**	**F**	**MS**	**F**	**MS**	**F**	**MS**	**F**	**MS**
Yield	41.86	76.92^b^	6.03	11.08^b^	0.84	1.55^b^	2.14	3.94^b^	0.54
Spike number	314,633.1	83.64^b^	150,272.5	39.95^b^	7997.3	2.13^b^	17,120.0	4.55^b^	3761.9
Kernel number per spike	2577.48	223.56^b^	346.23	30.03^b^	21.02	1.82^b^	50.74	4.40^b^	11.53
1000-Kernel weight	628.29	42.81^b^	176.54	12.03^b^	21.79	1.49^b^	61.55	4.19^b^	14.68
Fertility period	2364.39	1462.90^b^	7.70	4.77^b^	3.96	2.45^b^	5.60	3.47^b^	1.62
Seedling habit	48.28	140.87^b^	11.86	4.06^b^	0.75	2.19^b^	0.85	2.48^b^	0.34
Maturity performance	29.92	23.85^b^	2.73	2.17^a^	3.03	2.41^b^	2.08	1.66^b^	1.26
Plant height	3014.21	332.04^b^	386.66	42.59^b^	14.91	1.64^b^	119.70	13.19^b^	9.08
Lodging degree	32.08	29.44^b^	16.35	15.00^b^	3.31	3.04^b^	5.11	4.68^b^	1.09
Lodging area	14,605.02	29.02^b^	8344.08	16.58^b^	1465.04	2.91^b^	2512.85	4.99^b^	503.21
Winter injury	16.07	140.22^b^	1.22	10.60^b^	0.39	3.44^b^	0.20	1.76^b^	0.12
Spring cold injury	84.90	589.11^b^	0.35	2.39^a^	0.58	3.99^b^	0.13	0.91^b^	0.14
Powdery mildew resistance	75.38	177.73^b^	7.97	18.79^b^	0.92	2.16^b^	1.72	4.04^b^	0.42
Sharp eyespot resistance	113.65	104.20^b^	0.63	0.58	1.25	1.14	1.08	0.99	1.09
*Fusarium* head blight resistance	51.23	55.60^b^	4.40	4.78^b^	2.11	2.29^b^	1.74	1.89^b^	0.92
Leaf rust incidence	236,397.87	614.70^b^	1744.56	4.54^b^	987.98	2.57^b^	529.50	1.38^b^	384.58
Leaf rust severity	111,846.03	299.81^b^	1587.24	4.26^b^	794.07	2.13^b^	565.29	1.52^b^	373.05
Leaf rust reaction type	36.00	59.09^b^	11.32	18.58^b^	1.42	2.32^b^	2.33	3.82^b^	0.61
Stripe rust incidence	17,888.96	51.45^b^	1736.83	5.00^b^	871.22	2.51^b^	370.78	1.07	347.71
Stripe rust severity	4822.40	11.485^b^	1049.97	2.505^b^	195.06	0.46	485.54	1.16	420.07
Stripe rust reaction type	33.86	52.57^b^	2.16	3.35^b^	0.31	0.48	0.70	1.09	0.64

^a^ and ^b^ indicate significant at levels of 5% and 1%, respectively

### Characteristics of each subgroup

Subgroup I contained 31 cultivars, including Tianning 18 (Additional file 2: Table S2), which were characterized by high spike numbers, lodging, susceptibility to powdery mildew, small grain size, and low yield. Cultivars in this subgroup had an average of 646.3 spikes/m^2^ and an average 1,000-kernel weight of 44.25 g. Additionally, the highest degree of lodging and lodging area were recorded in this subgroup. The severity of powdery mildew was 3.44, which was the highest among all subgroups. The yield was 7.86 t/ha (Table 3), which was the second lowest among the subgroups (0.59 t/ha lower than that recorded in the subgroup with the highest yield). The parent cultivars used for breeding in this subgroup were of mixed origin. We observed that 29 of the 31 cultivars in this subgroup shared some characteristics with cultivars in the other subgroups, among which Xumai 457 shared 63% of its characteristics with members of four subgroups (Fig. 3, Additional file 3: Table S3).

**Table 3:** Comparison of the means of different subgroups for each trait

**Trait**	**Subgroup**									
	**I**	**II**	**III**	**IV**	**V**	**VI**	**VII**	**VIII**	**IX**	**X**
Number of cultivars	31	27	23	21	10	36	7	52	19	17
Yield (t/ha)	7.86c	7.98bc	8.45a	7.93bc	8.08b	8.02bc	7.60d	7.99bc	8.08b	8.08b
Spike number (spikes/m^2^)	646.3a	631.4a	607.4bc	613.1b	633.0a	595.7 cd	484.5e	595.5 cd	589.1d	607.7bc
Kernel number per spike	32.39 cd	31.51d	33.04bc	31.62d	31.99d	33.06bc	39.51a	33.64b	33.19bc	33.52b
1000-Kernel weight (g)	44.25d	45.71c	46.85ab	47.56a	45.46c	46.82ab	45.47c	45.98bc	47.43a	46.28bc
Fertility period (d)	231.3 cd	231.2cde	231.4bc	231.2cde	231.7ab	231.3 cd	230.9e	231.4bc	231.0de	231.8ab
Seedling habit	3.18abc	2.99d	3.29ab	3.19abc	2.63e	3.07 cd	3.12bcd	3.24ab	3.21abc	3.34a
Maturity performance	2.94b	2.64b	2.78b	2.65b	2.91b	2.72b	3.24a	2.80b	2.76b	2.75b
Plant height (cm)	82.16bc	79.62e	82.66b	84.17a	83.60a	81.50 cd	81.18d	79.80e	81.03d	82.22bc
Lodging degree	2.36a	1.81cdq	1.64def	1.99bc	2.13ab	1.56efg	1.33 g	2.02bc	1.38 fg	1.89bcd
Lodging area (%)	29.14a	16.42bc	11.83 cd	19.83b	20.05b	9.78d	6.02d	20.70b	7.16d	17.18bc
Winter injury	2.12cde	2.05def	2.23ab	2.08cde	2.04ef	2.14 cd	1.96f	2.23ab	2.17bc	2.28a
Spring cold injury	1.82ab	1.73ab	1.80ab	1.86a	1.67b	1.88a	1.67b	1.88a	1.84a	1.88a
Powder mildew resistance	3.44a	3.26b	2.79e	3.15bc	3.04 cd	2.91de	3.29ab	3.20bc	2.85e	3.28ab
*Fusarium* head blight resistance	3.60abc	3.64abc	3.88a	3.00d	3.57abc	3.32 cd	3.38bcd	3.47abc	3.75ab	3.24 cd
Leaf rust incidence (%)	54.03bcd	60.67ab	46.16de	56.11abc	43.83e	51.34cde	62.38a	53.81bcd	54.04bcd	46.47de
Leaf rust severity (%)	29.33abc	34.22ab	19.87d	28.30abcd	22.37 cd	21.50 cd	34.57a	26.38bcd	23.91 cd	23.00 cd
Leaf rust reaction type	3.11c	3.6a	2.7ef	3.15c	2.64f	2.97 cd	3.37b	3.11c	3.12c	2.88de
Stripe rust incidence (%)	7.90 cd	7.13 cd	4.46d	5.60d	6.00 cd	15.83abc	21.43ab	12.84bcd	24.21a	7.79 cd
Stripe rust severity (%)	7.74b	8.70b	5.65b	8.81b	13.00ab	13.96ab	22.86a	14.09ab	20.66a	14.56ab
Stripe rust reaction type	1.94 cd	1.91 cd	1.83d	1.93 cd	1.95 cd	2.28abc	2.64a	2.19bcd	2.39ab	2.06bcd

The same letter after the average values of different subgroups indicates that there is no significant difference based on Duncan's new repolarization test, and different letters indicate that the difference is significant

Subgroup II contained 27 cultivars, including Fumai 188 and Chuangmai 11 (Additional file 2: Table S2), which were characterized by dwarf stalks, a high spike number, a low grain number, cold tolerance, and low resistance to leaf rust. The average plant height of cultivars in this subgroup was 79.62 cm, which was the lowest among all subgroups (4.55 cm lower than that of the subgroup with the highest value). The average spike number was 631.4 spikes/m^2^, approximately 14.9 spikes/m^2^ fewer than the highest spike number recorded in any subgroup and 146.9 spikes/m^2^ more than the lowest recorded in any subgroup. The average kernel number per spike was 31.51 (Table 3). The cultivars in this subgroup had the lowest degree of winter and spring cold injury and the highest incidence and severity of leaf rust among the subgroups. The Aikang 58 and Zhoumai cultivars were the primary parents for breeding in this subgroup. Among the 27 cultivars in this subgroup, 14 (52%) were developed using Aikang 58, whereas 12 (44%) cultivars were developed using Zhoumai cultivars. Population structure analysis further demonstrated that 24 of the 27 cultivars in the subgroup shared 34.2 ~ 62.2% of their characteristics with members of other subgroups (Fig. 2, Additional file 3: Table S3).

Subgroup III comprised 23 cultivars, such as Keyu 368 (Additional file 2: Table S2). The three elements of yield (spike number, kernel number per spike, and 1,000-kernel weight) for these cultivars were related, and the cultivars were disease resistant; therefore, these cultivars had high yield. Cultivars in this subgroup had an average spike number of 607.4 spikes/m^2^, an average kernel number per spike of 33.04 grains, an average 1,000-kernel weight of 46.85 g, and an average yield of 8.45 t/ha (Table 3). Additionally, cultivars in this subgroup had high resistance to powdery mildew, leaf rust, and stripe rust, in addition to moderate plant height. Analysis of the cross combinations showed that Zhoumai cultivars were used for developing 15 of the 27 cultivars in this subgroup. Population structure analysis further demonstrated that 14 of the 23 cultivars shared some of their characteristics with members of other subgroups (Fig. 3). However, none shared more than 50.8% of their characteristics with other subgroups (Additional file 3: Table S3).

Subgroup IV comprised 21 cultivars, including Xinhuamai 818 (Additional file 2: Table S2), which were characterized by tall stalks, large grain size, and high stripe rust resistance. Cultivars in this subgroup displayed an average plant height of 84.17 cm and 1,000-kernel weight of 47.56 g (Table 3), which was the highest among all subgroups. Additionally, cultivars in this group had the second highest resistance to stripe rust among all subgroups. Analysis of the cross combinations of the cultivars showed that Zhoumai 16 and Zhoumai 18, as well as other Zhoumai cultivars, were used for breeding in this subgroup. Population structure analysis showed that the 21 cultivars of this subgroup shared 10.8 ~ 70.6% of their characteristics with members of other subgroups (Fig. 3, Additional file 3: Table S3).

Subgroup V comprised 10 cultivars, including Shengmai 102 (Additional file 2: Table S2), which were characterized by tall stalks, cold resistance, and poor lodging resistance. Cultivars in this subgroup showed an average plant height of 83.60 cm (Table 3), which was the second highest among all subgroups. The cultivars showed the highest resistance to freezing damage and leaf and stripe rust. Analysis of the cross combinations showed that cultivars from other provinces, primarily Shandong, were used as one or both parents for breeding all cultivars in this subgroup. Population structure analysis showed that 6/10 cultivars shared 5.4 ~ 59.9% of their characteristics with members of the other subgroups (Fig. 3, Additional file 3: Table S3).

Subgroup VI comprised 36 cultivars, including Xianmai 522 (Additional file 2: Table S2), which were characterized by large grain size and high lodging resistance. Cultivars in this subgroup had an average 1000-kernel weight of 46.82 g, which was only 0.74 g lower than that of the subgroup with the highest value (47.56 g, Table 3). The cultivars had a low lodging degree and area. Zhoumai 16 and Zhoumai 18 were used as parents for breeding 50% of the cultivars in this subgroup. Population structure analysis showed that 26 of the 36 cultivars shared 1.4 ~ 60.4% of their characteristics with members of other subgroups (Fig. 3, Additional file 3: Table S3).

Subgroup VII comprised 7 cultivars, including Jiamai 99 (Additional file 2: Table S2), which were characterized by large spikes, high grain numbers, lodging resistance, cold tolerance, high susceptibility to stripe rust, and low yield. Cultivars in this subgroup exhibited the highest severity of stripe rust and lowest spike number (484.5 spikes/m^2^), average lodging degree, average lodging area, degree of freezing injury, and average yield (7.60 t/ha). The average kernel number per spike was 39.51 grains (Table 3). Zhoumai cultivars were used as parents for breeding, whereas many different cultivars were used as the other parent. Population structure analysis showed that two of the seven cultivars shared 40.6 ~ 74.4% of their characteristics with members of other subgroups (Fig. 3, Additional file 3: Table S3).

Subgroup VIII included 52 cultivars, including Zhongxin 18 (Additional file 2: Table S2), which were characterized by dwarf stalks and poor resistance to sharp eyespot; however, the three yield elements (spike number, kernel number per spike, and 1,000-kernel weight) were associated. Cultivars in this group had an average plant height of 79.80 cm, which was the second lowest of all subgroups and only 0.18 cm higher than that of the subgroup with the lowest height. The average spike number was 595.5 spikes/m^2^, the average kernel number per spike was 33.64 grains, and the average 1,000-kernel weight was 45.98 g (Table 3). The resistance to sharp eyespot in the cultivars was the worst (2.74 grade). In the breeding process of the cultivars in this subgroup, the parents used were more diverse, and the Zhoumai series was used most often. In total, 45 of the 52 cultivars shared 3.4 ~ 73.2% of their characteristics with members of the other subgroups (Fig. 3, Additional file 3: Table S3).

Subgroup IX comprised 19 cultivars, including Chuangxin 106 (Additional file 2: Table S2), which were characterized by early maturity, large grain size, lodging resistance, and severe *Fusarium* head blight and stripe rust. The average fertility period was 231.0 days, which was the shortest among all subgroups, showing early maturity. The average 1,000-grain weight (47.43 g) was the second highest in each subgroup. The cultivars had the lowest average lodging degree and lodging area. *Fusarium* head blight resistance was the worst (3.75 grade) among all subgroups (Table 3). This subgroup also had the highest incidence of and strongest reaction to stripe rust. Popular cultivars in Henan Province were used as parents for breeding in this subgroup. Additionally, three of 19 cultivars shared 13.4 ~ 53.8% of characteristics with members of the other subgroups (Fig. 3, Additional file 3: Table S3).

Subgroup X comprised 17 cultivars, including Heyu 1 (Additional file 2: Table S2), which were characterized by associated yield components (spike number, kernel number per spike, and 1,000-kernel weight), long fertility periods, poor maturity performance, and low cold tolerance. The cultivars had an average spike number of 607.7 spikes/m^2^, an average kernel number per spike of 33.52 kernels, and an average 1,000-kernel weight of 46.28 g, which were relatively balanced. The average fertility period was 231.8 d, which was the longest observed among all subgroups. The seedling habit was grade 3.34 (Table 3)**,** which was the highest among all subgroups and showed weak springiness. The average degree of injury caused by low temperatures during winter and spring was 2.28 and 1.88, respectively, indicating poor cold tolerance. Most parents used for breeding were local and Zhoumai cultivars. In total, 12 of the 17 cultivars shared 31.0 ~ 66.0% of their characteristics with members of the other subgroups (Fig. 3, Additional file 3: Table S3).

## Discussion

### Genetic diversity of modern wheat cultivars in Henan Province

During the breeding process, owing to the different preferences for breeding units for specific traits, there is an inherent level of genetic differentiation between breeding cultivars, allowing them to have unique characteristics. In addition, the geographical location of breeding units varies, and there are certain differences in ecological conditions. The selection pressure caused by ecological conditions can also affect the characteristics of the bred varieties. Yang et al. stated that “the temperature in Henan generally decreases from southern to northern latitudes, but the ecological factors in different regions provide different selection pressures and have shaped plant architecture and growing season” (41). Genetic diversity analysis showed that 61.3% of the SNPs had high genetic differentiation (Fst > 0.2); 69.7% had high genetic differentiation in subgenome B, whereas 52.0% and 56.6% had high genetic differentiation in subgenomes A and D, respectively. The analysis of nucleotide diversity showed that the nucleotide diversity of subgenome B was relatively high, with an average π value of 3.91E-5; the nucleotide diversity of subgenome D was the lowest, with an average π value of 2.44E-5. Compared with that reported by Hao et al. (14), the π value was slightly smaller, which may be related to the use of different materials and SNP typing chips. These results indicate that after years of repeated selection, there is a higher degree of genetic differentiation with low nucleotide diversity in newly bred cultivars in Henan Province. Hence, it is difficult to breed breakthrough cultivars by crossing these cultivars with each other. However, the “best” cultivars can be used as parents for hybridization with foreign varieties or to introduce superior genes from related plants to breed new cultivars with breakthrough yield potential, disease resistance, stress resistance, and quality.

### Analysis of backbone parents for wheat breeding in Henan Province

PCA showed that Zhoumai cultivars were the most commonly used parents for breeding the modern wheat cultivars present in Henan Province. Zhoumai cultivars are new wheat cultivars bred by the Zhoukou Academy of Agricultural Sciences since the 1980s and are widely planted in the Huang Huai wheat-producing region. Zhoumai cultivars are high yielding and disease resistant. According to statistical data published in 2016 (internal data of Henan Province Seed Management Station, China), five of the six most cultivated wheat cultivars (> 333 kha) in Henan Province possess genetic materials from Zhoumai cultivars. Among 11 wheat cultivars with an annual planting area of 67 ~ 333 kha, six possess genetic materials from Zhoumai. Since 2011, 64% of the semiwinter wheat cultivars certified by Henan Province have been found to possess genetic materials from Zhoumai, whereas 67% of the semiwinter wheat cultivars certified by the state have genetic materials from Zhoumai. Thus, it is evident that Zhoumai cultivars are the most important backbone parents in wheat cross-breeding in Henan Province.

The second most commonly used parent for breeding modern cultivars was Aikang 58, which is a high-yield dwarf wheat variety with multiple resistance and wide adaptability. The largest annual planting area is more than 3 million ha, and the cumulative planting area is more than 20 million ha. Many breeding units have bred 128 approved wheat cultivars with Aikang 58 as a parent.

The third type is high-quality parents, which differ from those prevalent more than 20 years ago, although most breeding work requires further improvement of yields and stress resistance. However, increasing attention has been given to improving quality, which has become one of the main wheat breeding objectives in Henan Province. Nevertheless, quality should not compromise yield, and vice versa. Indeed, cultivars with favorable quality but poor yield will not be accepted by farmers owing to profitability issues. Therefore, breeders must attempt to breed cultivars with an acceptable combination of quality, yield, and stress resistance.

The fourth most common parent cultivars were those from Shandong, which is adjacent to Henan Province and has similar ecological and climatic conditions. Thus, Shandong cultivars have good adaptability in Henan Province, as well as good quality and great yield potential. Hence, Shandong wheat cultivars have made significant contributions to the genetic diversity of modern Henan wheat cultivars.

Based on analysis of the cross combinations of the cultivars corresponding to the ten elements with the largest absolute coefficient values for each PC in PCA, it can be seen that the parents used in wheat cross-breeding in Henan Province are quite similar, with a relatively homogenous genetic background. Thus, considering the low genetic diversity of modern wheat cultivars in Henan Province, new genetic materials from other countries or regions should be used to improve diversity and production performance, while genomic techniques, such as hybridization, genetic modification, and cloning, could be employed to enhance the genetic diversity and performance of wheat cultivars.

### Population structure of modern wheat cultivars in Henan Province

The objective evaluation of cultivars and classification of subgroups are important for the rational distribution and application of cultivars. Previously, wheat populations were clustered based on the phenotypic traits of cultivars. However, most phenotypic traits of wheat are quantitative traits, which are easily affected by environmental conditions. The expression of these phenotypic traits varies with location and time. For the same population, even if the same group of phenotypic traits and classification methods are used, the clustering results will differ if the test time or location differs. However, PCA clustering based on genotype diversity is more stable and reliable, as the results are not affected by the test time or location. With the advancement of molecular biology techniques, researchers have identified loci of interest using genetic markers. In the present study, the Affymetrix Axiom Wheat660K SNP chip was employed to examine the genetic diversity and population structure of 243 modern wheat cultivars in Henan Province. After performing a series of quality control steps, 395,783 SNP markers were retained, and 243 cultivars were assigned to ten subgroups. Except for susceptibility to powdery mildew and sharp eyespot, the remaining phenotypes significantly differed among subgroups. However, members of the subgroups shared certain characteristics and traits.

Subgroup III had the highest yield and disease resistance. The cultivars in this subgroup, as well as those in other subgroups with various beneficial characteristics, including high yield, high quality, stress resistance, and disease resistance, can be popularized in production following regional testing, production testing, and approval. Of course, these cultivars can also be used as parents, and superior cultivars can be bred through further genetic improvement. For instance, to increase the number of spikes per unit area, it is important to focus on using cultivars in subgroups I and II as parents. Moreover, to increase grain weight, cultivars in subgroups IV, VI, and IX would be beneficial as parents. To reduce plant height, cultivars in subgroups II and VIII should be considered parents, and to improve cold tolerance, those in subgroups II and V can be used. Furthermore, to improve lodging resistance, cultivars in subgroups VI, VII, and IX would represent effective parents. Finally, if large panicles and numerous grains are desired, the cultivars in subgroup VII should be used as parents. Hence, collectively, the findings of this study provide comprehensive insights for the selection of optimal cultivars with excellent comprehensive traits or specific breeding objectives in Henan Province and other regions.

## Conclusions

In summary, the genetic diversity and population structure of 243 modern wheat cultivars in Henan Province were analyzed by SNP genotyping and phenotypic data of related traits in this study. A higher degree of genetic differentiation was observed among different cultivars. That is, SNPs with a high degree of genetic differentiation (Fst > 0.2) accounted for 61.3%, whereas those with a moderate degree (Fst = 0.05 ~ 0.2) accounted for 33.4% of all SNPs. Based on the analysis of cross combinations of cultivars corresponding to the ten elements with the largest absolute coefficients for each PC in PCA, Zhoumai cultivars were identified as the main parent of the tested cultivars, followed by Aikang 58, high-quality cultivars, and Shandong cultivars. Through population genetic structure analysis, these cultivars were further divided into ten subgroups, each with its own distinct characteristics and unique utilization value. Hence, these results not only contribute to the objective evaluation and utilization of the tested varieties but also further our current understanding regarding the current challenges affecting wheat cultivars in Henan Province. This inspires us to scientifically formulate breeding objectives and strategies to improve the efficiency and speed of the breeding process. The primary limitation of this paper is the near exclusive inclusion of semiwinter cultivars. Thus, future cultivar investigations should be extended to those prevalent in areas with weak spring and spring production and larger areas.

## Methods

### Plant materials

Field experiments were conducted at Xihua (33°45ʹN, 114°24ʹE), Luohe (33°25ʹN, 113°29ʹE), Xuchang (34°03ʹN, 113°51ʹE), Xinxiang (35°09ʹN, 113°48ʹE), Wenxian (35°06ʹN, 113°05ʹE), Huixian (35°21ʹN, 113°48ʹE), and Nanle (36°13ʹN, 115°40ʹE) in Henan Province in 2015–2016. The experimental materials were 243 bread wheat (*Triticum aestivum* L.) cultivars included in the Comparative Test of Wheat Varieties in Henan Province, and Zhoumai 18 was used as the control cultivar. The tested cultivars were randomly arranged. To reduce experimental error, the experiment was based on the interval contrast design, a control was set every six cultivars, 210 basic seedlings/m^2^ were planted, and the area of each plot was at least 13 m^2^. Sowing occurred on approximately October 10, and harvest occurred in early June of the following year. Field management procedures, including fertilization, soil preparation, sowing, and irrigation, were carried out according to general field experimentation protocols.

### Phenotyping of plant traits

According to the standard NY/T 1301–2007 guidelines (wheat variety regional test record items and standards) issued by the Ministry of Agriculture, China, we examined the expression of 21 traits, including seedling habit and plant height, in each cultivar at all 7 test sites. The observation and recording standards for each trait are as follows:

- Harvest and yield measurement: Harvest and threshing were performed in a timely manner during the wax ripening stage. After air drying and sun drying, the yield was measured.
- Spike number: Before maturity, 3 evenly emerged sample points (1 m length segments) were selected within the plot, and the effective spike number was determined and converted to spikes/m^2^.
- Kernel number per spike: Before harvest, 50 single spikes were randomly harvested from each plot, mixed and threshed, the total kernel number was determined, and the average grains per spike was calculated.
- 1000-Kernel weight: One thousand seeds were randomly weighed (unit: g) twice, and the average value was taken (if the difference between the two averages exceeded 0.5 g, this step was reperformed), accurate to one decimal place.
- Fertility period: The fertility period was measured as the number of days from emergence to maturity.
- Seedling habit: Seedling habit was observed during the tillering period, with three levels: creeping, semicreeping and upright; expressed as 1, 2, and 3, respectively.
- Maturity performance: This trait was ranked as good, medium, and poor based on stem and leaf yellowing; expressed as 1, 3, and 5, respectively.
- Plant height: Plant height was measured from the ground to the top of the spike, excluding awns, in centimeters.
- Lodging resistance: This trait was measured twice as initial lodging and final lodging, recording the lodging date, degree and area, summarized by the final lodging data. The lodging area was the percentage of lodged area relative to the plot area. The lodging degree was recorded at five levels: no lodging; slight lodging, plant tilting angle less than or equal to 30°; moderate lodging, tilting angle 30° to 45° (inclusive of 45°); heavy lodging, tilting angle 45° to 60° (inclusive of 60°); and severe lodging, tilting angle above 60°; expressed as 1, 2, 3, 4, and 5, respectively.
- Cold resistance: According to the above ground freeze damage recorded in both the overwintering and spring stages, the plants were divided into five levels: no freeze damage, leaf tips turned yellow from freezing, half of the leaf blade frozen until dead, entire leaf blade withered, and plant or most tillers frozen until dead; expressed as 1, 2, 3, 4, and 5, respectively.
- Powdery mildew: This trait was recorded as five levels during the outbreak period during heading: no visible symptoms on leaves, disease on basal leaves, lesions spread to middle leaves, lesions spread to flag leaves, and lesions spread to spikes and awns; expressed as 1, 2, 3, 4, and 5, respectively.
- Sharp eyespot: Stem peeling was observed during the peak period of onset after full heading, with this trait recorded as five levels: no symptoms, leaf sheath infected but not penetrating the stem, lesions penetrating the stem but not to more than 1/4 (including 1/4) of the stem circumference, lesions penetrating the stem to between 1/4 and 3/4 (including 3/4) of the stem circumference, and lesions penetrating the stem to more than 3/4 of the circumference; expressed as 1, 2, 3, 4, and 5, respectively.
- *Fusarium* head blight: The severity of infection on the spikelets was visually estimated, and this trait was recorded as five levels: no diseased spikes, 1/4 (including 1/4) or fewer spikelets diseased, 1/4 to 1/2 (including 1/2) of spikelets diseased, 1/2 to 3/4 (including 3/4) of spikelets diseased, and more than 3/4 of spikelets diseased; expressed as 1, 2, 3, 4, and 5, respectively.
- Rust diseases: Leaf rust and stripe rust were recorded in terms of prevalence, severity, and reaction type. Incidence was estimated as the percentage of diseased leaves relative to the total number of leaves. Severity was estimated as the percentage of the lesion distribution relative to the leaf (sheath, stem) area. Reaction type was divided into five levels: immune, completely symptomless, or with very small pale spots; highly resistant, leaves with yellow‒white dead spots or very small spore masses, surrounded by obvious dead spots; moderately resistant, summer spore masses few and scattered, surrounded by fading green or dead spots; moderately susceptible, more summer spore masses, surrounded by fading green; and highly susceptible, many large summer spore masses, with no fading green surrounding; expressed as 1, 2, 3, 4, and 5, respectively.

### Statistical analysis

A joint analysis of variance was conducted on the results from each test location by phenotypic trait based on a fixed model. The total variation was partitioned among five factors: test locations, subgroups, test location × subgroup, cultivars within subgroup, and experimental error. Multiple comparisons between different subgroups were conducted using Duncan's new multiple-range test. Statistical calculations were implemented in SPSS 26.0.

### Genotyping of the tested cultivars

In this study, an improved sodium dodecyl sarcosine method [45, 46] was used to extract DNA from leaf tissue samples of the cultivars. The cultivars were genotyped with 630,517 SNP markers (http://wheat.pw.usda.gov/GG2/index.shtml) from the Affymetrix Axiom Wheat660K SNP chip at Beijing CapitalBio Technology Co., Ltd. This chip was developed by Jizeng Jia et al. at the Institute of Crop Sciences, Chinese Academy of Agricultural Sciences, and Affymetrix by sequencing 192 wheat varieties, including 60 modern cultivars, 72 local varieties, 30 wild one-grain varieties (*Triticum boeoticum*), and 30 varieties of *Aegilops tauschii*. Approximately 90% of the 630,517 SNP markers were polymorphic [42, 43]. The genotype data were analyzed using GenomeStudio software and exported and saved as text files.

### Quality control of genotype data

SNP markers with a heterozygosity rate > 10%, sample detection rate < 90%, and minimum allele frequency < 0.02 were excluded using PLINK V1.07 software [22]. Finally, 395,783 high-quality SNPs were retained for further analysis.

### Calculation of the population differentiation index and nucleotide diversity

Raw data for the 395,783 SNPs were converted into VCF format. The population differentiation index (Fst) and nucleotide diversity index (π) were calculated using VCFtools V4.1 software [47]. The calculation window was 100 Kb, and the step size was 100 Kb. The CMplot package of R software was used to plot the chromosome and physical position information of the SNPs.

### Principal Component Analysis (PCA)

PCA is a statistical method that transforms multiple interrelated characteristic indexes into a few independent comprehensive characteristic indexes. It not only uses the eigenvalue to reflect the relative contribution rate of each principal component but also uses the coefficients of each element of the principal component (eigenvector) to analyze the main features of each principal component. It is also an effective method for visualizing the distribution of different genotypes. PCA of the 243 wheat cultivars in Henan Province was performed using the GAPIT 3.0 software package developed by Cornell University in R studio [44, 48, 49].

### Population structure analysis

We compared the genetic structure of the samples using ADMIXTURE V1.3 software [17] based on a dataset containing 395,783 high-quality SNPs. *K* values ranging from 1 to 20 were tested until the minimum validation error was achieved. In total, 20 independent runs were performed for each *K* value.

### Supplementary Information

**Additional file 1: Table S1.** Cross combinations of the cultivars corresponding to the ten elements with the largest absolute values of the coefficients of their elements corresponding to top 8 principal components.

**Additional file 2: Table S2.** Tested cultivars from each subgroup.

**Additional file 3: Table S3.** The probability that the tested cultivars belong to a subgroup.

## Data Availability

The datasets generated and/or analysed during the current study are available in the ScienceDB repository, 10.57760/sciencedb.o00123.00001 or https://cstr.cn/31253.11.sciencedb.o00123.00001.

## Abbreviations

SNP: Single nucleotide polymorphism
PCA: Principal component analysis
