# Supplementary material for: Genetic diversity and population structure of modern wheat (Triticum aestivum L.) cultivars in Henan Province of China based on SNP markers
Source: BMC Plant Biol. 2023 Nov 4;23:542. doi: 10.1186/s12870-023-04537-9 (PMC10625233; doi:10.1186/s12870-023-04537-9)
Supplement: Supplementary file 1 — Additional file 1: Table S1. Cross combinations of the cultivars corresponding to the ten elements with the largest absolute values of the coefficients of their elements corresponding to top 8 principal components. [file 12870_2023_4537_MOESM1_ESM.docx]

**Table S1** Cross combinations of the cultivars corresponding to the ten elements with the largest absolute values of the coefficients of their elements corresponding to top 8 principal components.

| **Principal component** | **Cultivar** | **Cross combination** |
| --- | --- | --- |
| PC1 | Zhongle 8 | Tongzhoumai 916 × Bainong 207 |
|  | Xunhe 183 | (Zhoumai 22 × Bainong 64) × Zhoumai 16 |
|  | Kaimai 26 | Hengguan 35 × Liuhudali |
|  | Hemai 6 | Hemai 026 × Aikang 58 |
|  | Anyumai 18 | An 0444 × Yumai 50 |
|  | Kelinmai 969 | (Zhoumai16 × Yanzhan 4110) × Aikang 58 |
|  | Wohua 066 | (Zhoumai 22 × Neixiang 185) × Heze 17 |
|  | Shunmai 299 | Huayu 198 × Zhoumai 16 |
|  | Chuangxin 116 | Aizhou 11 × Luo 9908 |
|  | Zimai 615 | 03 Zhong 35 × Yujiao 5 |
| PC2 | Hefeng 3 | Zhoumai 16 dasui × Zhoumai 20 |
|  | Xinhuamai 818 | Zhoumai 13 × Bainong 4805 |
|  | Hangmai 8 | Tongzhoumai 916 × Bainong 207 |
|  | Xuyan 2 | Zhoumai 22 × Ruixing 989 |
|  | Zhengxin 758 | (Zhoumai 13 × Yumai 57) × Yumai 54 |
|  | Tianmai 119 | Zhoumai 18 × Zhoumai 22 |
|  | Meng 615 | (Neixiang 188 × Zhoumai12) × Huapei 3 |
|  | Yuyan 168 | Yumai 52 × Luomai 4 |
|  | Xinmai 68 | Zhongmai 12 × Aikang 58 |
|  | Zhongle 9 | Yumai 49 × Changzi 05-18A |
| PC3 | Shenzhou 209 | (Bian A × 921) × (Aikang 58 × Zhoumai 19) |
|  | Boyu 866 | (Aizao 781 × Yumai 2) × Xianyanchaodasui |
|  | Xianmai 15 | Aikang 58 × Xinong 979 |
|  | Xinxuan 17 | Aikang 58 × Xin 516 |
|  | Chuangxing 6 | (Aikang 58 × Zhengmai 366) × Zhoumai 16 |
|  | Jinmai 1 | JN 518-6 × JN 88-1998 |
|  | Xunmai 118 | (Kaimai 18 × Jimai 20) × (Yanzhan 4110 × Xinmai 18) |
|  | Caizhi 204 | 03(114)-3-1-3 × Zhoumai 16 |
|  | Dapingyuan 18 | (Yumai 34 × Yumai 49) × Zhoumai 13 |
|  | Zhengmai 516 | Aikang 58 × Jimai 22 |
| PC4 | Pingnongyan 3 | Zhoumai 16 × Pingmai 8 |
|  | Jinfeng 205 | Zhoumai 12 × Yumai 49 |
|  | Huimai 216 | Zhoumai 16 × Xinmai 16 |
|  | Jiyanmai 7 | Zhengmai 366 × Ji 05-5053 |
|  | Tianlaoda 1 | Zhengyumai 9987 × (Huapei 5 × Aikang 58) |
|  | Neile 268 | Zhoumai 18 × 03 Fan 20-0-0-12-3 |
|  | Hongmai 186 | Aikang 58 × Zhoumai 16 |
|  | Chuangxing 26 | Zhoumai 16 × Aikang 58 |
|  | Zhaofeng 668 | Zhoumai 16 × (Handan 6172 × Aikang 58)F_1_ |
|  | Zhengda 3087 | Zhoumai 16 × Zhengmai 9023 |
| PC5 | Luo 1807 | (A-3 × Zhoumai 22) × Zhoumai 22 |
|  | Zhengpinmai 24 | Yumai 34-6 × Yutong 194 |
|  | Shunmai 8 | PH 82-2 × Yumai 34 |
|  | Jiangmai 816 | Xumai 954 × Yan 2801 |
|  | Lunxuan 163 | Luoxin 998 × 119-1 |
|  | Qiule 2126 | Zhengmai 98 × Zhoumai 16 |
|  | TH 161 | TH 077 × Hangmai 0903 |
|  | Jingjiumai 11 | Yanzhan 4110 × Aikang 58 |
|  | Zhongfengmai 2 | 9910-7 × Zhoumai 18 |
|  | Xuke 732 | Aikang 58 × Zhoumai 18 |
| PC6 | SM110 | (Zhoumai 22 × Aikang 58) × Zhengyumai 9987 |
|  | Xinmai 68 | Zhongmai 12 × Aikang 58 |
|  | Tianmai 119 | Zhoumai 18 × Zhoumai 22 |
|  | Yuyan 168 | Yumai 52 × Luomai 4 |
|  | Meng 615 | (Neixiang 188 × Zhoumai 12) × Huapei 3 |
|  | Xuyan 2 | Zhoumai 22 × Ruixing 989 |
|  | Zhengxin758 | (Zhoumai 13 × Yumai 57) × Yumai 54 |
|  | Hangmai 8 | Tongzhoumai 916 × Bainong 207 |
|  | Hefeng 3 | Zhoumai 16 dasui × Zhoumai 20 |
|  | Xinhuamai 818 | Zhoumai 13 × Bainong 4805 |
| PC7 | Zimai 615 | 03 Zhong 35 × Yujiao 5 |
|  | Chuangxin 116 | Aizhou 11 × Luo 9908 |
|  | Shunmai 299 | Huayu 198 × Zhoumai 16 |
|  | Wohua 066 | (Zhoumai 22 × Neixiang 185) × Heze 17 |
|  | Jinfeng 216 | (Zhoumai 16 × Yumai 10) × Aikang 58 |
|  | Fannong 1 | Zhongfan 4 × Xinong 979 |
|  | Weinong 208 | Lankaoaizao 8 × Zhoumai 22 |
|  | Kelinmai 969 | (Zhoumai 16 × Yanzhan 4110) × Aikang 58 |
|  | Luomai 166 | Zhoumai 18 × Bainong 66 |
|  | TH 161 | TH 077 × Hangmai 0903 |
| PC8 | Shenhua 208 | Aikang 58 × 04 Zhong 36 |
|  | Xunmai 118 | (Kaimai 18 × Jimai 20) × (Yanzhan 4110 × Xinmai 18) |
|  | Jinmai 1 | JN 518-6 × JN 88-1998 |
|  | Gengmai 256 | Jimai 22 × Zhoumai 24 |
|  | Haozhuangjia 1 |  |
|  | Luyan 260 | Luyuan 502 × Jimai 22 |
|  | Ximai 505 | (Shi 4185 × Laizhou 95021) × Liangxing 99 |
|  | Shengmai 102 | Shannong 2149 × Liangxing 619 |
|  | Taifeng 11 | Yumai 25 × Liangxing 66 |
|  | Yanfeng 712 | Zhoumai 16 × Liangxing 66 |
