# Supplementary material for: Genetic diversity and population structure of modern wheat (Triticum aestivum L.) cultivars in Henan Province of China based on SNP markers
Source: BMC Plant Biol. 2023 Nov 4;23:542. doi: 10.1186/s12870-023-04537-9 (PMC10625233; doi:10.1186/s12870-023-04537-9)
Supplement: Supplementary file 2 — Additional file 2: Table S2. Tested cultivars from each subgroup. [file 12870_2023_4537_MOESM2_ESM.docx]

**Table S2** Tested cultivars from each subgroup.

| **Subgroup** | **Number of cultivars** | **Cultivars** |
| --- | --- | --- |
| Ⅰ | 31 | Yimai 8, Aomai 18, Tianning 18, Luomai 2, Xingyu 7, Yufeng 1366, Junmai 612, Shengyuan 928, Jiamai 6, Jinsui 8, Zhengnong 5222, Junmai 667, Fanmai 536, Xiza 5, Wanmai 99, Qinmai 158, Baiqiang 1201, Kaimai 27, Yanmai 888,Xinmai 38, Xinyoumai 2, Xunsui 188, Danmai 108, Zhengmai 516, Jinwoye 1, Shangmai 162, Yanmai 26, Dapingyuan 007, Neihuang 6, Sanhe 1, Xumai 457 |
| Ⅱ | 27 | Boyu 866, Xianmai 15, Shenzhou 209, Xinxuan 17, Xunong 618, Aifeng 338, Caizhi 204, Luyuan 502, Jinmai 109, Xunmai 118, Chuangxing 6, Jinmai 1, Zhoukang 918, Jinmai 14, Dongfanghong 6, Fumai 188, Bainong 219, Wenliang 1, Xuke 732, Chuangmai 11, Defeng 108, Xinmai 37, Wenyu 019,Yanbo 306, Saidemai 7, Dapingyuan 18, Cunmai 19 |
| Ⅲ | 23 | Xinmai 68, Zhengxin 758, Hefeng 3, Hangmai 8, Tianmai 119, Meng 615, Xuyan 2, Yuyan 168, Xinhuamai 818, Zhonglemai 9, Keyu 368, Zimai 627, Bainong 1309, Guangtai 213, Bainong 1306, Guangtai 369, Cunmai 20, Jimai 210, Huayu 126, Yunong 019, Xuyou 46, Liming 28, Shaomai 25 |
| Ⅳ | 21 | Nongda 399, Zhongkenmai 7, Maifeng 9, Nongfeng 111, Tianlaoda 3, Taixue 30, Xu 331, Yufeng 6, Yanke 316, Xianhong 169, L668, Liangmai 958, Fengbao 8, Zhengmai 082, Hengda 58, Luomai 718, Zhumai 706, Tongfeng 736, Qunximai 11, Hengmai 18, Changmai 13 |
| Ⅴ | 10 | Shengmai 102, Yanfeng 712, Ximai 505, Taifeng 11, Luyan 260, Gengmai 237, Gengmai 256, Haozhuangjia 1, Zhengmai 518, Yanmai 988 |
| Ⅵ | 36 | Jinfeng 216, Lunxuan 167, Chuangxin 116, Luomai 166, Fannong 1, Kelinmai 969, Wohua 066, Zimai 615, Weinong 208, Shunmai 299, Yingman 180, Yanmai 68, Fanyumai 18, Xuke 158, Xianmai 522, Xuyan 3, Tianmin 688, Yumai 117, Yingmai 182, Yufeng 2, Xiangmai 1123, Xuanmai 6, Jumai 66, Ximai 329, Liangyuan A6, Zhengda 101, Jinzhan 638, Mengnong 1, Minfeng 296, Shenhua 208, Danmai 118, Lifu 05, Xinxuan 16, Bomai 118, Zhenmai 5, Xianmai 521 |
| Ⅶ | 7 | Fengmai 53, Ningnong 718, Kun 169, SM 110, Fengmai 52, Xuke 682, Jiamai 99 |
| Ⅷ | 52 | Xunhuo 183, Kaimai 26, Hemai 6, Zhongle 8, Fannong 3, Qiangmai 29, Anyumai 18, Ruisen 218, Lunxuan 162, Lunxuan 169, Fengtian 18, Xinzhi 519, Yanmai 9719, Yunong 805, Zhengmai 1869, Zhouyumai 36, Jinmai 18, Zhongying 012, Xinmai 8, Hemai 181, Hangmai 6, Yulong 1325, Jingyumai 1, Yunong169, Xun 5366, Jingkemai 6, Jiamei 8, Wenmai 29, Changshengmai 1, Zhongxin 18, Xingnong 168, Yongfengnong 2, Nongfeng 8210, Jinying 18, Songmai 518, Jinyan 5, Jinmai 108, Luomai 32, Zhongmai 10, Xinmai 12, Hongmai 618, Hemai 2, Huaichuan 361, Wenyuan 0528, Zhongmai 108, Xianyuan 988, Caiyuan 1, Xuke 877, Huayu 3568,Zhengnong 06118, Pingan 0602, Jinchengmai 12 |
| Ⅸ | 19 | Zhengda 3087, Zhaofeng 668, Fengmai 10, Chuangxing 26, Yufeng 1, Neile 268, Pingnongyan 3, Hongtaiyang 2, Tianlaoda 1, Xinyanmai 98, Hongmai 186, Huimai 216, Jiyanmai 7, Jinfeng 205, Taihemai 3, Zhengke 6, Huayan 328, Nongda 2018, Chuangxin 106 |
| Ⅹ | 17 | Luo 1807, Lunxuan 163, Zhengpinmai 24, Shunmai 8, Qiule 2126, Jingjiumai 11, TH161, Heyu 1, Fanmai 533, Yunong 99, Jiyanmai 10, Jinchengmai 10, Jiangmai 816, Tianhe 6, Yunong 804, Zhongfengmai 2, Xinong 18 |
