# Supplementary material for: Genetic diversity and population structure of modern wheat (Triticum aestivum L.) cultivars in Henan Province of China based on SNP markers
Source: BMC Plant Biol. 2023 Nov 4;23:542. doi: 10.1186/s12870-023-04537-9 (PMC10625233; doi:10.1186/s12870-023-04537-9)
Supplement: Supplementary file 3 — Additional file 3: Table S3. The probability that the tested cultivars belong to a subgroup. [file 12870_2023_4537_MOESM3_ESM.docx]

**Table S3** The probability that the tested cultivars belong to a subgroup.

| **Subgroup** | | **Cultivar name** | **Q1** | **Q2** | **Q3** | **Q4** | **Q5** | **Q6** | **Q7** | **Q8** | **Q9** | **Q10** |
| --- | --- | --- | --- | --- | --- | --- | --- | --- | --- | --- | --- | --- |
| Ⅰ | Aomai 18 | 0.9999 | 0.0000 | 0.0000 | 0.0000 | 0.0000 | 0.0000 | 0.0000 | 0.0000 | 0.0000 | 0.0000 |  |
| Ⅰ | Baiqiang 1201 | 0.5927 | 0.1172 | 0.0000 | 0.1172 | 0.1549 | 0.0000 | 0.0178 | 0.0000 | 0.0000 | 0.0000 |  |
| Ⅰ | Dapingyuan 007 | 0.4998 | 0.0000 | 0.0000 | 0.4605 | 0.0396 | 0.0000 | 0.0000 | 0.0000 | 0.0000 | 0.0000 |  |
| Ⅰ | Danmai 108 | 0.5535 | 0.0000 | 0.0000 | 0.0000 | 0.0000 | 0.0000 | 0.0000 | 0.4464 | 0.0000 | 0.0000 |  |
| Ⅰ | Fanmai 536 | 0.6150 | 0.0000 | 0.0000 | 0.0000 | 0.0000 | 0.0000 | 0.0000 | 0.3779 | 0.0000 | 0.0071 |  |
| Ⅰ | Jiamai 6 | 0.6885 | 0.0000 | 0.0000 | 0.0000 | 0.3114 | 0.0000 | 0.0000 | 0.0000 | 0.0000 | 0.0000 |  |
| Ⅰ | Jinsui 8 | 0.6745 | 0.0387 | 0.0000 | 0.0000 | 0.2867 | 0.0000 | 0.0000 | 0.0000 | 0.0000 | 0.0000 |  |
| Ⅰ | Jinwoye 1 | 0.5220 | 0.0000 | 0.0195 | 0.3691 | 0.0646 | 0.0000 | 0.0000 | 0.0000 | 0.0000 | 0.0247 |  |
| Ⅰ | Junmai 612 | 0.7471 | 0.0000 | 0.0094 | 0.1031 | 0.1275 | 0.0000 | 0.0129 | 0.0000 | 0.0000 | 0.0000 |  |
| Ⅰ | Junmai 667 | 0.6150 | 0.0000 | 0.0000 | 0.0000 | 0.0000 | 0.0000 | 0.0000 | 0.2322 | 0.0000 | 0.1528 |  |
| Ⅰ | Xunsui 188 | 0.5566 | 0.0000 | 0.0000 | 0.0000 | 0.0000 | 0.0000 | 0.0364 | 0.0013 | 0.0000 | 0.4057 |  |
| Ⅰ | Kaimai 27 | 0.5927 | 0.0000 | 0.0748 | 0.0000 | 0.0000 | 0.0000 | 0.0000 | 0.3324 | 0.0000 | 0.0000 |  |
| Ⅰ | Luomai 2 | 0.8667 | 0.0000 | 0.0799 | 0.0000 | 0.0354 | 0.0000 | 0.0000 | 0.0000 | 0.0000 | 0.0179 |  |
| Ⅰ | Neihuang 6 | 0.4974 | 0.0000 | 0.0000 | 0.0000 | 0.0507 | 0.0000 | 0.0000 | 0.4519 | 0.0000 | 0.0000 |  |
| Ⅰ | Qinmai 158 | 0.5982 | 0.1620 | 0.0000 | 0.0000 | 0.1685 | 0.0000 | 0.0713 | 0.0000 | 0.0000 | 0.0000 |  |
| Ⅰ | Sanhe 1 | 0.4747 | 0.0000 | 0.0000 | 0.0000 | 0.0000 | 0.0000 | 0.0000 | 0.0935 | 0.0000 | 0.4318 |  |
| Ⅰ | Shangmai 162 | 0.5213 | 0.0000 | 0.0302 | 0.0000 | 0.0000 | 0.0000 | 0.0000 | 0.0839 | 0.0000 | 0.3645 |  |
| Ⅰ | Shengyuan 928 | 0.7131 | 0.0000 | 0.0000 | 0.0000 | 0.2676 | 0.0000 | 0.0192 | 0.0000 | 0.0000 | 0.0000 |  |
| Ⅰ | Tianning 18 | 0.8752 | 0.0000 | 0.0000 | 0.0045 | 0.0000 | 0.0000 | 0.1202 | 0.0000 | 0.0000 | 0.0000 |  |
| Ⅰ | Wanmai 99 | 0.6013 | 0.0000 | 0.0297 | 0.0000 | 0.0168 | 0.0000 | 0.0000 | 0.3394 | 0.0000 | 0.0128 |  |
| Ⅰ | Xiza 5 | 0.6153 | 0.0426 | 0.0000 | 0.0000 | 0.2287 | 0.0000 | 0.0287 | 0.0188 | 0.0000 | 0.0658 |  |
| Ⅰ | Xinmai 38 | 0.5657 | 0.4342 | 0.0000 | 0.0000 | 0.0000 | 0.0000 | 0.0000 | 0.0000 | 0.0000 | 0.0000 |  |
| Ⅰ | Xinyoumai 2 | 0.5603 | 0.4396 | 0.0000 | 0.0000 | 0.0000 | 0.0000 | 0.0000 | 0.0000 | 0.0000 | 0.0000 |  |
| Ⅰ | Xingyu 7 | 0.8497 | 0.0000 | 0.0000 | 0.1503 | 0.0000 | 0.0000 | 0.0000 | 0.0000 | 0.0000 | 0.0000 |  |
| Ⅰ | Xumai 457 | 0.3702 | 0.1617 | 0.0000 | 0.2410 | 0.0000 | 0.0000 | 0.0000 | 0.1230 | 0.0000 | 0.1040 |  |
| Ⅰ | Yanmai 26, | 0.5057 | 0.0000 | 0.0000 | 0.0000 | 0.0000 | 0.0000 | 0.0000 | 0.4942 | 0.0000 | 0.0000 |  |
| Ⅰ | Yanmai 888 | 0.5742 | 0.0000 | 0.0000 | 0.0000 | 0.2889 | 0.0000 | 0.0558 | 0.0000 | 0.0083 | 0.0728 |  |
| Ⅰ | Yimai 8 | 0.9999 | 0.0000 | 0.0000 | 0.0000 | 0.0000 | 0.0000 | 0.0000 | 0.0000 | 0.0000 | 0.0000 |  |
| Ⅰ | Yufeng 1366 | 0.8264 | 0.0000 | 0.0000 | 0.1261 | 0.0000 | 0.0000 | 0.0000 | 0.0475 | 0.0000 | 0.0000 |  |
| Ⅰ | Zhengmai 516 | 0.5501 | 0.4498 | 0.0000 | 0.0000 | 0.0000 | 0.0000 | 0.0000 | 0.0000 | 0.0000 | 0.0000 |  |
| Ⅰ | Zhengnong 5222 | 0.6419 | 0.0000 | 0.0000 | 0.2033 | 0.0991 | 0.0000 | 0.0000 | 0.0557 | 0.0000 | 0.0000 |  |
| Ⅱ | Aifeng 338 | 0.0000 | 0.6314 | 0.0000 | 0.0000 | 0.1879 | 0.0000 | 0.0758 | 0.0000 | 0.0000 | 0.1047 |  |
| Ⅱ | Bainong 21 9 | 0.0000 | 0.5239 | 0.0712 | 0.2002 | 0.0000 | 0.0000 | 0.0000 | 0.2046 | 0.0000 | 0.0000 |  |
| Ⅱ | Boyu 866 | 0.0000 | 0.9999 | 0.0000 | 0.0000 | 0.0000 | 0.0000 | 0.0000 | 0.0000 | 0.0000 | 0.0000 |  |
| Ⅱ | Caizhi 204 | 0.0000 | 0.6142 | 0.0000 | 0.0000 | 0.0000 | 0.0000 | 0.0000 | 0.3858 | 0.0000 | 0.0000 |  |
| Ⅱ | Chuangmai 11 | 0.0000 | 0.5202 | 0.0000 | 0.0000 | 0.0000 | 0.0000 | 0.0000 | 0.4797 | 0.0000 | 0.0000 |  |
| Ⅱ | Chuangxing 6 | 0.4248 | 0.5613 | 0.0000 | 0.0000 | 0.0138 | 0.0000 | 0.0000 | 0.0000 | 0.0000 | 0.0000 |  |
| Ⅱ | Dapingyuan 18 | 0.4686 | 0.4698 | 0.0238 | 0.0000 | 0.0376 | 0.0000 | 0.0000 | 0.0000 | 0.0000 | 0.0000 |  |
| Ⅱ | Defeng 108 | 0.0000 | 0.5146 | 0.0000 | 0.0000 | 0.0000 | 0.0000 | 0.0000 | 0.4854 | 0.0000 | 0.0000 |  |
| Ⅱ | Dongfanghong 6 | 0.0000 | 0.5356 | 0.0648 | 0.1266 | 0.0000 | 0.0000 | 0.0479 | 0.0010 | 0.0000 | 0.2241 |  |
| Ⅱ | Cunmai 19 | 0.2791 | 0.3778 | 0.0000 | 0.0000 | 0.1536 | 0.0000 | 0.0822 | 0.1072 | 0.0000 | 0.0000 |  |
| Ⅱ | Fumai 188 | 0.0000 | 0.5278 | 0.4721 | 0.0000 | 0.0000 | 0.0000 | 0.0000 | 0.0000 | 0.0000 | 0.0000 |  |
| Ⅱ | Jinmai 109 | 0.0000 | 0.5843 | 0.0000 | 0.0000 | 0.0000 | 0.0000 | 0.0000 | 0.0000 | 0.0000 | 0.4156 |  |
| Ⅱ | Jinmai 1 | 0.0000 | 0.5583 | 0.0000 | 0.0000 | 0.4416 | 0.0000 | 0.0000 | 0.0000 | 0.0000 | 0.0000 |  |
| Ⅱ | Jinmai 14 | 0.0000 | 0.5436 | 0.0244 | 0.4319 | 0.0000 | 0.0000 | 0.0000 | 0.0000 | 0.0000 | 0.0000 |  |
| Ⅱ | Xunmai 118 | 0.0000 | 0.5635 | 0.0000 | 0.0000 | 0.4365 | 0.0000 | 0.0000 | 0.0000 | 0.0000 | 0.0000 |  |
| Ⅱ | Luyuan 502 | 0.1103 | 0.5918 | 0.0021 | 0.0723 | 0.0000 | 0.0000 | 0.0000 | 0.2235 | 0.0000 | 0.0000 |  |
| Ⅱ | Saidemai 7 | 0.0000 | 0.4881 | 0.0868 | 0.0880 | 0.0000 | 0.0000 | 0.0000 | 0.0000 | 0.0000 | 0.3371 |  |
| Ⅱ | Shenzhou 209 | 0.0000 | 0.9999 | 0.0000 | 0.0000 | 0.0000 | 0.0000 | 0.0000 | 0.0000 | 0.0000 | 0.0000 |  |
| Ⅱ | Wenliang 1 | 0.0000 | 0.5225 | 0.0331 | 0.0000 | 0.0000 | 0.0000 | 0.0000 | 0.0000 | 0.0000 | 0.4444 |  |
| Ⅱ | Wenyu 019 | 0.0000 | 0.5143 | 0.0000 | 0.0000 | 0.0000 | 0.0000 | 0.0000 | 0.4857 | 0.0000 | 0.0000 |  |
| Ⅱ | Xianmai 15 | 0.0000 | 0.9999 | 0.0000 | 0.0000 | 0.0000 | 0.0000 | 0.0000 | 0.0000 | 0.0000 | 0.0000 |  |
| Ⅱ | Xinmai 37 | 0.0000 | 0.5137 | 0.0000 | 0.0000 | 0.0000 | 0.0000 | 0.0000 | 0.0000 | 0.0000 | 0.4862 |  |
| Ⅱ | Xinxuan 17 | 0.0119 | 0.6583 | 0.0000 | 0.0669 | 0.1735 | 0.0000 | 0.0671 | 0.0224 | 0.0000 | 0.0000 |  |
| Ⅱ | Xuke 732 | 0.0000 | 0.5198 | 0.0000 | 0.0000 | 0.0000 | 0.0000 | 0.0000 | 0.0000 | 0.0000 | 0.4801 |  |
| Ⅱ | Xunong 618 | 0.0000 | 0.6327 | 0.0000 | 0.0000 | 0.2151 | 0.0000 | 0.0649 | 0.0000 | 0.0000 | 0.0871 |  |
| Ⅱ | Yanbo 306 | 0.1168 | 0.5095 | 0.0000 | 0.0000 | 0.1000 | 0.0000 | 0.0318 | 0.2418 | 0.0000 | 0.0000 |  |
| Ⅱ | Zhoukang 918 | 0.0000 | 0.5554 | 0.0000 | 0.0000 | 0.0000 | 0.0000 | 0.0199 | 0.3644 | 0.0000 | 0.0602 |  |
| Ⅲ | Bainong 1306 | 0.0000 | 0.0057 | 0.5512 | 0.3034 | 0.0000 | 0.0000 | 0.0000 | 0.1396 | 0.0000 | 0.0000 |  |
| Ⅲ | Bainong 1309 | 0.3415 | 0.0000 | 0.5811 | 0.0480 | 0.0000 | 0.0000 | 0.0000 | 0.0293 | 0.0000 | 0.0000 |  |
| Ⅲ | Cunmai 20 | 0.0000 | 0.0000 | 0.5317 | 0.0000 | 0.0843 | 0.0000 | 0.0000 | 0.3549 | 0.0000 | 0.0291 |  |
| Ⅲ | Guangtai 213 | 0.3134 | 0.0000 | 0.5576 | 0.0000 | 0.0553 | 0.0000 | 0.0737 | 0.0000 | 0.0000 | 0.0000 |  |
| Ⅲ | Guangtai 369 | 0.1120 | 0.0100 | 0.5348 | 0.0759 | 0.0908 | 0.0000 | 0.1136 | 0.0000 | 0.0000 | 0.0628 |  |
| Ⅲ | Hangmai 8 | 0.0000 | 0.0000 | 0.9999 | 0.0000 | 0.0000 | 0.0000 | 0.0000 | 0.0000 | 0.0000 | 0.0000 |  |
| Ⅲ | Hefeng 3 | 0.0000 | 0.0000 | 0.9999 | 0.0000 | 0.0000 | 0.0000 | 0.0000 | 0.0000 | 0.0000 | 0.0000 |  |
| Ⅲ | Huayu 126 | 0.0000 | 0.0000 | 0.5121 | 0.2604 | 0.0000 | 0.0000 | 0.0000 | 0.2274 | 0.0000 | 0.0000 |  |
| Ⅲ | Jimai 210 | 0.0000 | 0.0196 | 0.5285 | 0.4452 | 0.0000 | 0.0000 | 0.0066 | 0.0000 | 0.0000 | 0.0000 |  |
| Ⅲ | Keyu 368 | 0.2035 | 0.0000 | 0.7964 | 0.0000 | 0.0000 | 0.0000 | 0.0000 | 0.0000 | 0.0000 | 0.0000 |  |
| Ⅲ | Liming 28 | 0.3794 | 0.0000 | 0.4931 | 0.0000 | 0.1274 | 0.0000 | 0.0000 | 0.0000 | 0.0000 | 0.0000 |  |
| Ⅲ | Meng 615 | 0.0000 | 0.0000 | 0.9999 | 0.0000 | 0.0000 | 0.0000 | 0.0000 | 0.0000 | 0.0000 | 0.0000 |  |
| Ⅲ | Shaomai 25 | 0.0000 | 0.0000 | 0.4925 | 0.1202 | 0.0000 | 0.0000 | 0.0230 | 0.3186 | 0.0000 | 0.0456 |  |
| Ⅲ | Tianmai 119 | 0.0000 | 0.0000 | 0.9999 | 0.0000 | 0.0000 | 0.0000 | 0.0000 | 0.0000 | 0.0000 | 0.0000 |  |
| Ⅲ | Xinhuamai 818 | 0.0000 | 0.0000 | 0.9999 | 0.0000 | 0.0000 | 0.0000 | 0.0000 | 0.0000 | 0.0000 | 0.0000 |  |
| Ⅲ | Xinmai 68 | 0.0000 | 0.0000 | 0.9999 | 0.0000 | 0.0000 | 0.0000 | 0.0000 | 0.0000 | 0.0000 | 0.0000 |  |
| Ⅲ | Xuyan 2 | 0.0000 | 0.0000 | 0.9999 | 0.0000 | 0.0000 | 0.0000 | 0.0000 | 0.0000 | 0.0000 | 0.0000 |  |
| Ⅲ | Xuyou 46 | 0.0000 | 0.0194 | 0.4981 | 0.0000 | 0.0000 | 0.0000 | 0.0675 | 0.1611 | 0.2539 | 0.0000 |  |
| Ⅲ | Yunong 019 | 0.0000 | 0.4968 | 0.5031 | 0.0000 | 0.0000 | 0.0000 | 0.0000 | 0.0000 | 0.0000 | 0.0000 |  |
| Ⅲ | Yuyan 168 | 0.0000 | 0.0000 | 0.9999 | 0.0000 | 0.0000 | 0.0000 | 0.0000 | 0.0000 | 0.0000 | 0.0000 |  |
| Ⅲ | Zhengxin 758 | 0.0000 | 0.0000 | 0.9999 | 0.0000 | 0.0000 | 0.0000 | 0.0000 | 0.0000 | 0.0000 | 0.0000 |  |
| Ⅲ | Zhonglemai 9 | 0.0000 | 0.0000 | 0.8681 | 0.0000 | 0.0000 | 0.0000 | 0.0000 | 0.1319 | 0.0000 | 0.0000 |  |
| Ⅲ | Zimai 627 | 0.0000 | 0.0000 | 0.5859 | 0.0000 | 0.0000 | 0.0000 | 0.3623 | 0.0000 | 0.0000 | 0.0518 |  |
| Ⅳ | L668 | 0.0000 | 0.0000 | 0.0000 | 0.5711 | 0.0000 | 0.0000 | 0.0000 | 0.4288 | 0.0000 | 0.0000 |  |
| Ⅳ | Changmai 13 | 0.0000 | 0.2793 | 0.1108 | 0.2941 | 0.0000 | 0.0000 | 0.0378 | 0.0000 | 0.0000 | 0.2780 |  |
| Ⅳ | Fengbao 8 | 0.0000 | 0.0220 | 0.0231 | 0.5221 | 0.0000 | 0.0000 | 0.0000 | 0.4103 | 0.0000 | 0.0225 |  |
| Ⅳ | Hengmai 18 | 0.0000 | 0.0000 | 0.0000 | 0.3248 | 0.0965 | 0.0000 | 0.2688 | 0.0000 | 0.0000 | 0.3098 |  |
| Ⅳ | Hengda 58 | 0.0000 | 0.0283 | 0.0000 | 0.4495 | 0.2192 | 0.1207 | 0.1515 | 0.0027 | 0.0000 | 0.0280 |  |
| Ⅳ | Liangmai 958 | 0.4467 | 0.0000 | 0.0299 | 0.5233 | 0.0000 | 0.0000 | 0.0000 | 0.0000 | 0.0000 | 0.0000 |  |
| Ⅳ | Luomai 718 | 0.0012 | 0.0141 | 0.1374 | 0.4464 | 0.0000 | 0.0256 | 0.0335 | 0.0478 | 0.0061 | 0.2879 |  |
| Ⅳ | Maifeng 9 | 0.1714 | 0.0283 | 0.0672 | 0.7327 | 0.0004 | 0.0000 | 0.0000 | 0.0000 | 0.0000 | 0.0000 |  |
| Ⅳ | Nongda 399 | 0.0000 | 0.0000 | 0.0000 | 0.8919 | 0.0721 | 0.0000 | 0.0359 | 0.0000 | 0.0000 | 0.0000 |  |
| Ⅳ | Nongfeng 111 | 0.0000 | 0.0000 | 0.1677 | 0.7168 | 0.0000 | 0.0000 | 0.0000 | 0.0000 | 0.0000 | 0.1154 |  |
| Ⅳ | Xu 331 | 0.0000 | 0.0000 | 0.1079 | 0.6531 | 0.0000 | 0.0000 | 0.0000 | 0.2389 | 0.0000 | 0.0000 |  |
| Ⅳ | Qunximai 11 | 0.2542 | 0.0000 | 0.0000 | 0.3792 | 0.1886 | 0.0000 | 0.0508 | 0.0189 | 0.0000 | 0.1083 |  |
| Ⅳ | Taixue 30 | 0.0000 | 0.0000 | 0.0000 | 0.6550 | 0.0000 | 0.0000 | 0.0000 | 0.0000 | 0.0000 | 0.3450 |  |
| Ⅳ | Tianlaoda 3 | 0.0000 | 0.0000 | 0.0162 | 0.6768 | 0.0000 | 0.0000 | 0.0000 | 0.3070 | 0.0000 | 0.0000 |  |
| Ⅳ | Tongfeng 736 | 0.2879 | 0.0026 | 0.0148 | 0.3848 | 0.2220 | 0.0000 | 0.0878 | 0.0000 | 0.0000 | 0.0000 |  |
| Ⅳ | Xianhong 169 | 0.0439 | 0.0013 | 0.0486 | 0.5896 | 0.1670 | 0.0000 | 0.1495 | 0.0000 | 0.0000 | 0.0000 |  |
| Ⅳ | Yanke 316 | 0.0000 | 0.0000 | 0.0000 | 0.5992 | 0.3175 | 0.0000 | 0.0464 | 0.0369 | 0.0000 | 0.0000 |  |
| Ⅳ | Yufeng 6 | 0.0000 | 0.0000 | 0.1066 | 0.6008 | 0.0000 | 0.0000 | 0.0000 | 0.0000 | 0.0000 | 0.2925 |  |
| Ⅳ | Zhengmai 082 | 0.0000 | 0.0000 | 0.0000 | 0.5135 | 0.0000 | 0.0000 | 0.0000 | 0.0000 | 0.0000 | 0.4864 |  |
| Ⅳ | Zhongkenmai 7 | 0.0000 | 0.0819 | 0.0000 | 0.7376 | 0.1405 | 0.0000 | 0.0399 | 0.0000 | 0.0000 | 0.0000 |  |
| Ⅳ | Zhumai 706 | 0.0000 | 0.0634 | 0.3447 | 0.4266 | 0.0000 | 0.0000 | 0.1056 | 0.0000 | 0.0000 | 0.0596 |  |
| Ⅴ | Gengmai 237 | 0.0189 | 0.0000 | 0.0000 | 0.0000 | 0.7666 | 0.0000 | 0.0000 | 0.2144 | 0.0000 | 0.0000 |  |
| Ⅴ | Gengmai 256 | 0.0000 | 0.0000 | 0.0000 | 0.0000 | 0.6998 | 0.0000 | 0.0000 | 0.3002 | 0.0000 | 0.0000 |  |
| Ⅴ | Haozhuangjia 1 | 0.0000 | 0.0161 | 0.0000 | 0.2492 | 0.6446 | 0.0000 | 0.0000 | 0.0000 | 0.0000 | 0.0901 |  |
| Ⅴ | Luyan 260 | 0.0000 | 0.0000 | 0.0000 | 0.0000 | 0.9461 | 0.0000 | 0.0000 | 0.0000 | 0.0000 | 0.0538 |  |
| Ⅴ | Shengmai 102 | 0.0000 | 0.0000 | 0.0000 | 0.0000 | 0.9999 | 0.0000 | 0.0000 | 0.0000 | 0.0000 | 0.0000 |  |
| Ⅴ | Taifeng 11 | 0.0000 | 0.0000 | 0.0000 | 0.0000 | 0.9999 | 0.0000 | 0.0000 | 0.0000 | 0.0000 | 0.0000 |  |
| Ⅴ | Ximai 505 | 0.0000 | 0.0000 | 0.0000 | 0.0000 | 0.9999 | 0.0000 | 0.0000 | 0.0000 | 0.0000 | 0.0000 |  |
| Ⅴ | Yanmai 988 | 0.0551 | 0.1250 | 0.0000 | 0.2419 | 0.4010 | 0.0000 | 0.0219 | 0.0000 | 0.0000 | 0.1550 |  |
| Ⅴ | Yanfeng 712 | 0.0000 | 0.0000 | 0.0000 | 0.0000 | 0.9999 | 0.0000 | 0.0000 | 0.0000 | 0.0000 | 0.0000 |  |
| Ⅴ | Zhengmai 518 | 0.0000 | 0.0000 | 0.0000 | 0.3173 | 0.4219 | 0.0000 | 0.1862 | 0.0000 | 0.0000 | 0.0745 |  |
| Ⅵ | Bomai 118 | 0.2657 | 0.0000 | 0.0000 | 0.0000 | 0.0000 | 0.5861 | 0.0000 | 0.0000 | 0.1482 | 0.0000 |  |
| Ⅵ | Chuangxin 116 | 0.0000 | 0.0000 | 0.0000 | 0.0000 | 0.0000 | 0.9999 | 0.0000 | 0.0000 | 0.0000 | 0.0000 |  |
| Ⅵ | Danmai 118 | 0.1153 | 0.0000 | 0.0764 | 0.0131 | 0.0000 | 0.6290 | 0.0000 | 0.0000 | 0.1662 | 0.0000 |  |
| Ⅵ | Fannong 1 | 0.0000 | 0.0000 | 0.0000 | 0.0000 | 0.0000 | 0.9999 | 0.0000 | 0.0000 | 0.0000 | 0.0000 |  |
| Ⅵ | Fanyumai 18 | 0.0000 | 0.0000 | 0.0000 | 0.0000 | 0.0000 | 0.9374 | 0.0000 | 0.0000 | 0.0626 | 0.0000 |  |
| Ⅵ | Jinfeng 216 | 0.0000 | 0.0000 | 0.0000 | 0.0000 | 0.0000 | 0.9999 | 0.0000 | 0.0000 | 0.0000 | 0.0000 |  |
| Ⅵ | Jinzhan 638 | 0.0000 | 0.0000 | 0.0000 | 0.0000 | 0.0660 | 0.6755 | 0.0000 | 0.0000 | 0.2584 | 0.0000 |  |
| Ⅵ | Jumai 66 | 0.0000 | 0.0000 | 0.0000 | 0.0000 | 0.0004 | 0.8109 | 0.0000 | 0.0000 | 0.1886 | 0.0000 |  |
| Ⅵ | Kelinmai 969 | 0.0000 | 0.0000 | 0.0000 | 0.0000 | 0.0000 | 0.9999 | 0.0000 | 0.0000 | 0.0000 | 0.0000 |  |
| Ⅵ | Lifu 05 | 0.2630 | 0.0000 | 0.0000 | 0.0000 | 0.0000 | 0.6052 | 0.0000 | 0.0000 | 0.1317 | 0.0000 |  |
| Ⅵ | Liangyuan A6 | 0.0000 | 0.0488 | 0.0000 | 0.0000 | 0.0000 | 0.6960 | 0.0369 | 0.0000 | 0.2183 | 0.0000 |  |
| Ⅵ | Lunxuan 167 | 0.0000 | 0.0000 | 0.0000 | 0.0000 | 0.0000 | 0.9999 | 0.0000 | 0.0000 | 0.0000 | 0.0000 |  |
| Ⅵ | Luomai 166 | 0.0000 | 0.0000 | 0.0000 | 0.0000 | 0.0000 | 0.9999 | 0.0000 | 0.0000 | 0.0000 | 0.0000 |  |
| Ⅵ | Mengnong 1 | 0.0000 | 0.0147 | 0.0000 | 0.0000 | 0.0073 | 0.6679 | 0.1905 | 0.0000 | 0.1195 | 0.0000 |  |
| Ⅵ | Minfeng 296 | 0.0000 | 0.0080 | 0.0000 | 0.0154 | 0.1118 | 0.6544 | 0.0000 | 0.0000 | 0.2103 | 0.0000 |  |
| Ⅵ | Shenhua 208 | 0.2286 | 0.0000 | 0.0000 | 0.0000 | 0.0000 | 0.6307 | 0.0000 | 0.0000 | 0.1401 | 0.0005 |  |
| Ⅵ | Shunmai 299 | 0.0000 | 0.0000 | 0.0000 | 0.0000 | 0.0000 | 0.9999 | 0.0000 | 0.0000 | 0.0000 | 0.0000 |  |
| Ⅵ | Tianmin 688 | 0.0000 | 0.0080 | 0.0000 | 0.0000 | 0.0000 | 0.9057 | 0.0000 | 0.0000 | 0.0862 | 0.0000 |  |
| Ⅵ | Weinong 208 | 0.0000 | 0.0000 | 0.0000 | 0.0000 | 0.0000 | 0.9999 | 0.0000 | 0.0000 | 0.0000 | 0.0000 |  |
| Ⅵ | Wohua 066 | 0.0000 | 0.0000 | 0.0000 | 0.0000 | 0.0000 | 0.9999 | 0.0000 | 0.0000 | 0.0000 | 0.0000 |  |
| Ⅵ | Ximai 329 | 0.0000 | 0.0000 | 0.0000 | 0.0000 | 0.0521 | 0.7996 | 0.0000 | 0.0000 | 0.1483 | 0.0000 |  |
| Ⅵ | Xianmai 521 | 0.0000 | 0.0000 | 0.1096 | 0.0000 | 0.0000 | 0.3962 | 0.0122 | 0.0000 | 0.2792 | 0.2027 |  |
| Ⅵ | Xianmai 522 | 0.0723 | 0.0000 | 0.0000 | 0.0000 | 0.0000 | 0.9190 | 0.0000 | 0.0000 | 0.0086 | 0.0000 |  |
| Ⅵ | Xiangmai 1123 | 0.0000 | 0.0000 | 0.0167 | 0.0000 | 0.0000 | 0.8539 | 0.0000 | 0.0000 | 0.1293 | 0.0000 |  |
| Ⅵ | Xinxuan 16 | 0.0000 | 0.0416 | 0.0000 | 0.1385 | 0.0000 | 0.6002 | 0.0000 | 0.0000 | 0.2195 | 0.0000 |  |
| Ⅵ | Xuke 158, | 0.0000 | 0.0000 | 0.0000 | 0.0000 | 0.0000 | 0.9326 | 0.0000 | 0.0000 | 0.0673 | 0.0000 |  |
| Ⅵ | Xuyan 3 | 0.0000 | 0.0000 | 0.0000 | 0.0000 | 0.0553 | 0.9158 | 0.0000 | 0.0058 | 0.0230 | 0.0000 |  |
| Ⅵ | Xuanmai 6 | 0.0000 | 0.0000 | 0.0000 | 0.0000 | 0.0000 | 0.8432 | 0.0000 | 0.0000 | 0.1567 | 0.0000 |  |
| Ⅵ | Yanmai 68 | 0.0000 | 0.0000 | 0.0000 | 0.0000 | 0.0104 | 0.9487 | 0.0000 | 0.0000 | 0.0409 | 0.0000 |  |
| Ⅵ | Yingman 180 | 0.0000 | 0.0000 | 0.0000 | 0.0000 | 0.0000 | 0.9864 | 0.0000 | 0.0000 | 0.0135 | 0.0000 |  |
| Ⅵ | Yingmai 182 | 0.0000 | 0.0000 | 0.0000 | 0.0000 | 0.0000 | 0.8922 | 0.0000 | 0.0000 | 0.1077 | 0.0000 |  |
| Ⅵ | Yufeng 2 | 0.0000 | 0.0000 | 0.0000 | 0.0000 | 0.0141 | 0.8608 | 0.0000 | 0.0000 | 0.1250 | 0.0000 |  |
| Ⅵ | Yumai 117 | 0.0000 | 0.0000 | 0.0000 | 0.0000 | 0.0000 | 0.9010 | 0.0000 | 0.0000 | 0.0989 | 0.0000 |  |
| Ⅵ | Zhenmai 5 | 0.3908 | 0.0000 | 0.0000 | 0.0000 | 0.0000 | 0.4911 | 0.0000 | 0.0000 | 0.1180 | 0.0000 |  |
| Ⅵ | Zhengda 101 | 0.0000 | 0.0069 | 0.0000 | 0.0260 | 0.0000 | 0.6888 | 0.0491 | 0.0000 | 0.2291 | 0.0000 |  |
| Ⅵ | Zimai 615 | 0.0000 | 0.0000 | 0.0000 | 0.0000 | 0.0000 | 0.9999 | 0.0000 | 0.0000 | 0.0000 | 0.0000 |  |
| Ⅶ | SM 110 | 0.0000 | 0.0000 | 0.0000 | 0.0000 | 0.0000 | 0.0000 | 0.9999 | 0.0000 | 0.0000 | 0.0000 |  |
| Ⅶ | Fengmai 52, | 0.0000 | 0.0000 | 0.0000 | 0.0000 | 0.0000 | 0.0000 | 0.9999 | 0.0000 | 0.0000 | 0.0000 |  |
| Ⅶ | Fengmai 53 | 0.0000 | 0.0000 | 0.0000 | 0.0000 | 0.0000 | 0.0000 | 0.9999 | 0.0000 | 0.0000 | 0.0000 |  |
| Ⅶ | Jiamai 99 | 0.0779 | 0.0494 | 0.0000 | 0.1512 | 0.1923 | 0.0358 | 0.2560 | 0.2329 | 0.0000 | 0.0045 |  |
| Ⅶ | Kun 169 | 0.0000 | 0.0000 | 0.0000 | 0.0000 | 0.0000 | 0.0000 | 0.9999 | 0.0000 | 0.0000 | 0.0000 |  |
| Ⅶ | Ningnong 718 | 0.0000 | 0.0000 | 0.0000 | 0.0000 | 0.0000 | 0.0000 | 0.9999 | 0.0000 | 0.0000 | 0.0000 |  |
| Ⅶ | Xuke 682 | 0.0000 | 0.0000 | 0.1796 | 0.1110 | 0.0000 | 0.0000 | 0.5939 | 0.0000 | 0.0000 | 0.1154 |  |
| Ⅷ | Anyumai 18 | 0.0000 | 0.0000 | 0.0000 | 0.0000 | 0.0000 | 0.0000 | 0.0000 | 0.9999 | 0.0000 | 0.0000 |  |
| Ⅷ | Caiyuan 1 | 0.0000 | 0.0539 | 0.0000 | 0.1503 | 0.2326 | 0.0000 | 0.0000 | 0.4379 | 0.0000 | 0.1252 |  |
| Ⅷ | Changshengmai 1 | 0.0000 | 0.4372 | 0.0000 | 0.0000 | 0.0000 | 0.0000 | 0.0000 | 0.5628 | 0.0000 | 0.0000 |  |
| Ⅷ | Fannong 3 | 0.0000 | 0.0000 | 0.0000 | 0.0000 | 0.0000 | 0.0000 | 0.0000 | 0.9999 | 0.0000 | 0.0000 |  |
| Ⅷ | Fengtian 18 | 0.0000 | 0.0000 | 0.0000 | 0.0066 | 0.0778 | 0.0000 | 0.0000 | 0.8644 | 0.0000 | 0.0510 |  |
| Ⅷ | Hangmai 6 | 0.0000 | 0.0000 | 0.0000 | 0.0713 | 0.1923 | 0.0000 | 0.1226 | 0.6057 | 0.0081 | 0.0000 |  |
| Ⅷ | Hemai 181 | 0.0000 | 0.0000 | 0.0000 | 0.3564 | 0.0000 | 0.0000 | 0.0000 | 0.6305 | 0.0000 | 0.0131 |  |
| Ⅷ | Huaichuan 36 | 0.4059 | 0.0000 | 0.0000 | 0.0000 | 0.0000 | 0.0000 | 0.0000 | 0.4812 | 0.0000 | 0.1128 |  |
| Ⅷ | Hemai 6 | 0.0000 | 0.0000 | 0.0000 | 0.0000 | 0.0000 | 0.0000 | 0.0000 | 0.9999 | 0.0000 | 0.0000 |  |
| Ⅷ | Hongmai 618 | 0.0000 | 0.0486 | 0.0424 | 0.4119 | 0.0000 | 0.0000 | 0.0000 | 0.4970 | 0.0000 | 0.0000 |  |
| Ⅷ | Huayu 3568 | 0.0896 | 0.3635 | 0.0207 | 0.0230 | 0.0542 | 0.0000 | 0.0135 | 0.4329 | 0.0000 | 0.0025 |  |
| Ⅷ | Huaichuan 361 | 0.0000 | 0.0000 | 0.0789 | 0.4345 | 0.0000 | 0.0000 | 0.0000 | 0.4740 | 0.0125 | 0.0000 |  |
| Ⅷ | Jiamei 8 | 0.0000 | 0.4350 | 0.0000 | 0.0000 | 0.0000 | 0.0000 | 0.0000 | 0.5649 | 0.0000 | 0.0000 |  |
| Ⅷ | Jinchengmai 12 | 0.0360 | 0.1953 | 0.0000 | 0.1682 | 0.1628 | 0.0000 | 0.0000 | 0.2685 | 0.0000 | 0.1692 |  |
| Ⅷ | Jinmai 108 | 0.0000 | 0.4825 | 0.0000 | 0.0000 | 0.0000 | 0.0000 | 0.0000 | 0.5175 | 0.0000 | 0.0000 |  |
| Ⅷ | Jinmai 18 | 0.0000 | 0.0000 | 0.0000 | 0.2032 | 0.0000 | 0.0000 | 0.0000 | 0.7340 | 0.0000 | 0.0627 |  |
| Ⅷ | Jinying 18 | 0.0000 | 0.0000 | 0.3715 | 0.0000 | 0.0272 | 0.0000 | 0.0103 | 0.5456 | 0.0000 | 0.0453 |  |
| Ⅷ | Jinyan 5 | 0.0604 | 0.0000 | 0.0448 | 0.1161 | 0.0495 | 0.0000 | 0.0566 | 0.5213 | 0.0000 | 0.1513 |  |
| Ⅷ | Jingyumai 1 | 0.1298 | 0.0842 | 0.0000 | 0.0674 | 0.1283 | 0.0000 | 0.0000 | 0.5903 | 0.0000 | 0.0000 |  |
| Ⅷ | Jingkemai 6 | 0.0000 | 0.4292 | 0.0000 | 0.0000 | 0.0000 | 0.0000 | 0.0000 | 0.5707 | 0.0000 | 0.0000 |  |
| Ⅷ | Xun 5366 | 0.0000 | 0.0000 | 0.0000 | 0.3478 | 0.0000 | 0.0000 | 0.0000 | 0.5811 | 0.0000 | 0.0710 |  |
| Ⅷ | Xunhuo 183 | 0.0000 | 0.0000 | 0.0000 | 0.0000 | 0.0000 | 0.0000 | 0.0000 | 0.9999 | 0.0000 | 0.0000 |  |
| Ⅷ | Kaimai 26 | 0.0000 | 0.0000 | 0.0000 | 0.0000 | 0.0000 | 0.0000 | 0.0000 | 0.9999 | 0.0000 | 0.0000 |  |
| Ⅷ | Lunxuan 162 | 0.0000 | 0.0000 | 0.0000 | 0.0159 | 0.0000 | 0.0000 | 0.0000 | 0.9185 | 0.0000 | 0.0655 |  |
| Ⅷ | Lunxuan 169 | 0.0000 | 0.0000 | 0.0000 | 0.0000 | 0.0189 | 0.0000 | 0.0000 | 0.8941 | 0.0000 | 0.0869 |  |
| Ⅷ | Luomai 32 | 0.4769 | 0.0090 | 0.0000 | 0.0000 | 0.0000 | 0.0000 | 0.0000 | 0.5140 | 0.0000 | 0.0000 |  |
| Ⅷ | Nongfeng 8210 | 0.0000 | 0.0000 | 0.0000 | 0.0000 | 0.0000 | 0.0000 | 0.4445 | 0.5554 | 0.0000 | 0.0000 |  |
| Ⅷ | Pingan 0602 | 0.0000 | 0.0000 | 0.1967 | 0.2717 | 0.0000 | 0.0000 | 0.0170 | 0.2731 | 0.0000 | 0.2414 |  |
| Ⅷ | Qiangmai 29 | 0.0000 | 0.0000 | 0.0000 | 0.0000 | 0.0000 | 0.0000 | 0.0000 | 0.9999 | 0.0000 | 0.0000 |  |
| Ⅷ | Ruisen 218 | 0.0339 | 0.0000 | 0.0000 | 0.0000 | 0.0000 | 0.0000 | 0.0000 | 0.9660 | 0.0000 | 0.0000 |  |
| Ⅷ | Songmai 518 | 0.0000 | 0.0000 | 0.0000 | 0.0724 | 0.0000 | 0.0000 | 0.2996 | 0.5419 | 0.0000 | 0.0860 |  |
| Ⅷ | Wenmai 29 | 0.0000 | 0.0000 | 0.0000 | 0.0000 | 0.0000 | 0.0000 | 0.0000 | 0.5652 | 0.0000 | 0.4347 |  |
| Ⅷ | Wenyuan 0528 | 0.4343 | 0.0000 | 0.0000 | 0.0000 | 0.0999 | 0.0000 | 0.0000 | 0.4657 | 0.0000 | 0.0000 |  |
| Ⅷ | Xianyuan 988 | 0.0000 | 0.0000 | 0.0000 | 0.4074 | 0.0000 | 0.0000 | 0.1425 | 0.4500 | 0.0000 | 0.0000 |  |
| Ⅷ | Xinzhi 519 | 0.0000 | 0.0000 | 0.0000 | 0.0000 | 0.0000 | 0.0000 | 0.0000 | 0.8074 | 0.0000 | 0.1926 |  |
| Ⅷ | Xinmai 12 | 0.0000 | 0.4979 | 0.0000 | 0.0000 | 0.0000 | 0.0000 | 0.0000 | 0.5020 | 0.0000 | 0.0000 |  |
| Ⅷ | Xinmai 8 | 0.0000 | 0.0000 | 0.0000 | 0.0753 | 0.1095 | 0.0000 | 0.1094 | 0.7058 | 0.0000 | 0.0000 |  |
| Ⅷ | Xingnong 168, | 0.0000 | 0.4394 | 0.0000 | 0.0000 | 0.0000 | 0.0000 | 0.0000 | 0.5606 | 0.0000 | 0.0000 |  |
| Ⅷ | Xuke 877 | 0.3639 | 0.0000 | 0.0208 | 0.1746 | 0.0000 | 0.0000 | 0.0000 | 0.4333 | 0.0000 | 0.0074 |  |
| Ⅷ | Yanmai 9719 | 0.2004 | 0.0000 | 0.0000 | 0.0000 | 0.0000 | 0.0000 | 0.0000 | 0.7995 | 0.0000 | 0.0000 |  |
| Ⅷ | Yongfengnong 2 | 0.1370 | 0.0749 | 0.0000 | 0.0000 | 0.0719 | 0.0000 | 0.0975 | 0.5554 | 0.0000 | 0.0632 |  |
| Ⅷ | Yulong 1325 | 0.0000 | 0.0087 | 0.0000 | 0.1261 | 0.1464 | 0.0018 | 0.1251 | 0.5920 | 0.0000 | 0.0000 |  |
| Ⅷ | Yunong169 | 0.0000 | 0.0000 | 0.0000 | 0.0000 | 0.0000 | 0.0000 | 0.0000 | 0.5836 | 0.0000 | 0.4163 |  |
| Ⅷ | Yunong 805 | 0.0000 | 0.1108 | 0.1114 | 0.0000 | 0.0000 | 0.0000 | 0.0000 | 0.7778 | 0.0000 | 0.0000 |  |
| Ⅷ | Zhengmai 1869 | 0.0000 | 0.0000 | 0.0000 | 0.0000 | 0.1312 | 0.0000 | 0.0000 | 0.7498 | 0.0000 | 0.1189 |  |
| Ⅷ | Zhengnong 06118 | 0.0000 | 0.0000 | 0.0000 | 0.1218 | 0.0000 | 0.0000 | 0.3175 | 0.4193 | 0.0000 | 0.1414 |  |
| Ⅷ | Zhongle 8 | 0.0000 | 0.0000 | 0.0000 | 0.0000 | 0.0000 | 0.0000 | 0.0000 | 0.9999 | 0.0000 | 0.0000 |  |
| Ⅷ | Zhongxin 18 | 0.0000 | 0.4374 | 0.0000 | 0.0000 | 0.0000 | 0.0000 | 0.0000 | 0.5625 | 0.0000 | 0.0000 |  |
| Ⅷ | Zhongying 012 | 0.0000 | 0.0000 | 0.0000 | 0.0695 | 0.1096 | 0.0000 | 0.1053 | 0.7155 | 0.0000 | 0.0000 |  |
| Ⅷ | Xianyuan 988 | 0.0000 | 0.0000 | 0.0000 | 0.3951 | 0.0000 | 0.0000 | 0.1533 | 0.4516 | 0.0000 | 0.0000 |  |
| Ⅷ | Zhongmai 10 | 0.0379 | 0.0664 | 0.0070 | 0.0000 | 0.2261 | 0.0000 | 0.0367 | 0.5081 | 0.0187 | 0.0992 |  |
| Ⅷ | Zhouyumai 36 | 0.0000 | 0.0262 | 0.0000 | 0.0000 | 0.0733 | 0.0000 | 0.0557 | 0.7477 | 0.0000 | 0.0969 |  |
| Ⅸ | Chuangxin 106 | 0.0000 | 0.0000 | 0.0000 | 0.1444 | 0.0423 | 0.0000 | 0.0721 | 0.2768 | 0.4623 | 0.0020 |  |
| Ⅸ | Chuangxing 26 | 0.0000 | 0.0000 | 0.0000 | 0.0000 | 0.0000 | 0.0000 | 0.0000 | 0.0000 | 0.9999 | 0.0000 |  |
| Ⅸ | Fengmai 10 | 0.0000 | 0.0000 | 0.0000 | 0.0000 | 0.0000 | 0.0000 | 0.0000 | 0.0000 | 0.9999 | 0.0000 |  |
| Ⅸ | Hongtaiyang 2 | 0.0000 | 0.0000 | 0.0000 | 0.0000 | 0.0000 | 0.0000 | 0.0000 | 0.0000 | 0.9999 | 0.0000 |  |
| Ⅸ | Hongmai 186 | 0.0000 | 0.0000 | 0.0000 | 0.0000 | 0.0000 | 0.0000 | 0.0000 | 0.0000 | 0.9999 | 0.0000 |  |
| Ⅸ | Huayan 328 | 0.0000 | 0.0000 | 0.0000 | 0.0000 | 0.0000 | 0.1078 | 0.0000 | 0.0259 | 0.8662 | 0.0000 |  |
| Ⅸ | Huimai 216 | 0.0000 | 0.0000 | 0.0000 | 0.0000 | 0.0000 | 0.0000 | 0.0000 | 0.0000 | 0.9999 | 0.0000 |  |
| Ⅸ | Jiyanmai 7 | 0.0000 | 0.0000 | 0.0000 | 0.0000 | 0.0000 | 0.0000 | 0.0000 | 0.0000 | 0.9999 | 0.0000 |  |
| Ⅸ | Jinfeng 205 | 0.0000 | 0.0000 | 0.0000 | 0.0000 | 0.0000 | 0.0000 | 0.0000 | 0.0000 | 0.9999 | 0.0000 |  |
| Ⅸ | Neile 268 | 0.0000 | 0.0000 | 0.0000 | 0.0000 | 0.0000 | 0.0000 | 0.0000 | 0.0000 | 0.9999 | 0.0000 |  |
| Ⅸ | Nongda 2018, | 0.0000 | 0.0000 | 0.0000 | 0.0000 | 0.0000 | 0.1309 | 0.0000 | 0.0000 | 0.8574 | 0.0116 |  |
| Ⅸ | Pingnongyan 3 | 0.0000 | 0.0000 | 0.0000 | 0.0000 | 0.0000 | 0.0000 | 0.0000 | 0.0000 | 0.9999 | 0.0000 |  |
| Ⅸ | Taihemai 3 | 0.0000 | 0.0000 | 0.0000 | 0.0000 | 0.0000 | 0.0000 | 0.0000 | 0.0000 | 0.9999 | 0.0000 |  |
| Ⅸ | Tianlaoda 1 | 0.0000 | 0.0000 | 0.0000 | 0.0000 | 0.0000 | 0.0000 | 0.0000 | 0.0000 | 0.9999 | 0.0000 |  |
| Ⅸ | Xinyanmai 98 | 0.0000 | 0.0000 | 0.0000 | 0.0000 | 0.0000 | 0.0000 | 0.0000 | 0.0000 | 0.9999 | 0.0000 |  |
| Ⅸ | Yufeng 1 | 0.0000 | 0.0000 | 0.0000 | 0.0000 | 0.0000 | 0.0000 | 0.0000 | 0.0000 | 0.9999 | 0.0000 |  |
| Ⅸ | Zhaofeng 668 | 0.0000 | 0.0000 | 0.0000 | 0.0000 | 0.0000 | 0.0000 | 0.0000 | 0.0000 | 0.9999 | 0.0000 |  |
| Ⅸ | Zhengda 3087 | 0.0000 | 0.0000 | 0.0000 | 0.0000 | 0.0000 | 0.0000 | 0.0000 | 0.0000 | 0.9999 | 0.0000 |  |
| Ⅸ | Zhengke 6 | 0.0000 | 0.0000 | 0.0000 | 0.0000 | 0.0000 | 0.0000 | 0.0000 | 0.0000 | 0.9999 | 0.0000 |  |
| Ⅹ | TH161 | 0.0000 | 0.0489 | 0.0554 | 0.0000 | 0.0632 | 0.0000 | 0.1685 | 0.0000 | 0.0000 | 0.6640 |  |
| Ⅹ | Fanmai 533 | 0.3243 | 0.0000 | 0.0000 | 0.0000 | 0.0000 | 0.0000 | 0.0000 | 0.0961 | 0.0000 | 0.5795 |  |
| Ⅹ | Heyu 1 | 0.2759 | 0.0000 | 0.0000 | 0.0153 | 0.0000 | 0.0000 | 0.0286 | 0.0420 | 0.0000 | 0.6381 |  |
| Ⅹ | Jiyanmai 10 | 0.0000 | 0.4903 | 0.0000 | 0.0000 | 0.0000 | 0.0000 | 0.0000 | 0.0000 | 0.0000 | 0.5096 |  |
| Ⅹ | Jiangmai 816 | 0.0000 | 0.0000 | 0.0089 | 0.0000 | 0.0000 | 0.2160 | 0.0000 | 0.0000 | 0.2877 | 0.4873 |  |
| Ⅹ | Jinchengmai 10 | 0.0707 | 0.0000 | 0.0000 | 0.0000 | 0.0809 | 0.0000 | 0.0408 | 0.3117 | 0.0000 | 0.4960 |  |
| Ⅹ | Jingjiumai 11 | 0.0000 | 0.0585 | 0.0438 | 0.0000 | 0.0326 | 0.0000 | 0.1752 | 0.0000 | 0.0000 | 0.6898 |  |
| Ⅹ | Lunxuan 163 | 0.0000 | 0.0000 | 0.0000 | 0.0000 | 0.0000 | 0.0000 | 0.0000 | 0.0000 | 0.0000 | 0.9999 |  |
| Ⅹ | Luo 1807 | 0.0000 | 0.0000 | 0.0000 | 0.0000 | 0.0000 | 0.0000 | 0.0000 | 0.0000 | 0.0000 | 0.9999 |  |
| Ⅹ | Qiule 2126 | 0.0000 | 0.0000 | 0.0000 | 0.0000 | 0.0000 | 0.0000 | 0.0000 | 0.0000 | 0.0000 | 0.9999 |  |
| Ⅹ | Shunmai 8 | 0.0000 | 0.0000 | 0.0000 | 0.0000 | 0.0000 | 0.0000 | 0.0000 | 0.0000 | 0.0000 | 0.9999 |  |
| Ⅹ | Tianhe 6 | 0.0000 | 0.0000 | 0.0846 | 0.0000 | 0.0000 | 0.0000 | 0.0482 | 0.4119 | 0.0000 | 0.4552 |  |
| Ⅹ | Xinong 18 | 0.3185 | 0.0000 | 0.1009 | 0.1246 | 0.0000 | 0.0000 | 0.0000 | 0.1155 | 0.0000 | 0.3405 |  |
| Ⅹ | Yunong 804 | 0.0000 | 0.0748 | 0.0108 | 0.3339 | 0.0000 | 0.0000 | 0.0000 | 0.1338 | 0.0000 | 0.4467 |  |
| Ⅹ | Yunong 99 | 0.0000 | 0.0000 | 0.1429 | 0.0357 | 0.0000 | 0.0000 | 0.0000 | 0.2948 | 0.0000 | 0.5265 |  |
| Ⅹ | Zhengpinmai 24 | 0.0000 | 0.0000 | 0.0000 | 0.0000 | 0.0000 | 0.0000 | 0.0000 | 0.0000 | 0.0000 | 0.9999 |  |
| Ⅹ | Zhongfengmai 2 | 0.0000 | 0.1321 | 0.0893 | 0.1710 | 0.0349 | 0.0086 | 0.1711 | 0.0000 | 0.0000 | 0.3931 |  |
